# A Diguanylate Cyclase Acts as a Cell Division Inhibitor in a Two-Step Response to Reductive and Envelope Stresses

**DOI:** 10.1128/mBio.00822-16

**Published:** 2016-08-09

**Authors:** Hyo Kyung Kim, Rasika M. Harshey

**Affiliations:** Department of Molecular Biosciences and Institute of Cellular and Molecular Biology, University of Texas at Austin, Austin, Texas, USA

## Abstract

Cell division arrest is a universal checkpoint in response to environmental assaults that generate cellular stress. In bacteria, the cyclic di-GMP (c-di-GMP) signaling network is one of several signal transduction systems that regulate key processes in response to extra-/intracellular stimuli. Here, we find that the diguanylate cyclase YfiN acts as a bifunctional protein that produces c-di-GMP in response to reductive stress and then dynamically relocates to the division site to arrest cell division in response to envelope stress in *Escherichia coli*. YfiN localizes to the Z ring by interacting with early division proteins and stalls cell division by preventing the initiation of septal peptidoglycan synthesis. These studies reveal a new role for a diguanylate cyclase in responding to environmental change, as well as a novel mechanism for arresting cell division.

## IMPORTANCE

While the major role of c-di-GMP signaling is to control the decision to move freely or settle in a biofilm, recent studies show a broader range of output functions for c-di-GMP signaling. This work reports an unexpected second role for YfiN, a conserved diguanylate cyclase in Gram-negative bacteria, known to contribute to persistence in the host. We find that YfiN acts as a cell division inhibitor in response to envelope stress. Unlike known cell division inhibitors, the interaction of YfiN with cell division proteins retains the Z ring at the midcell but prevents septal invagination. The new function of YfiN not only emphasizes the versatility of c-di-GMP signaling but describes a novel mechanism for a cell division checkpoint.

## INTRODUCTION

Bacteria sense and respond to environmental signals through a variety of signaling pathways (1–3). Signaling through the second messenger cyclic di-GMP (c-di-GMP) is ubiquitous in bacteria, where its major role is to control the transition between motile and sessile lifestyles (1, 4). However, recent studies have shown that c-di-GMP also regulates other processes, including cell cycle progression, RNA metabolism, resistance to antimicrobial agents, virulence, and pathogenesis (5–9). Here, we report a new role for c-di-GMP signaling in *Escherichia coli*, where it inhibits cell division in response to envelope stress.

Cellular c-di-GMP levels are set by enzymes that synthesize and degrade this molecule in response to a variety of external and internal signals, both physical and metabolic. Diguanylate cyclases (DGCs), identifiable by a signature GGDEF active-site motif, produce c-di-GMP from GTP, while phosphodiesterases (PDEs), identifiable by an EAL (or HD-GYP) active-site motif, degrade c-di-GMP into pGpG (10). Most DGCs and PDEs harbor various N-terminal sensory input domains, which allow environmental and cellular signals to be integrated into the c-di-GMP signaling network. Although several environmental stimuli regulating DGCs and PDEs have been identified (11–13), the majority of input signals that orchestrate the c-di-GMP signaling network remain to be discovered, especially given the multiplicity of GGDEF/EAL domain proteins in single bacterial species (1).

**FIG 1:**
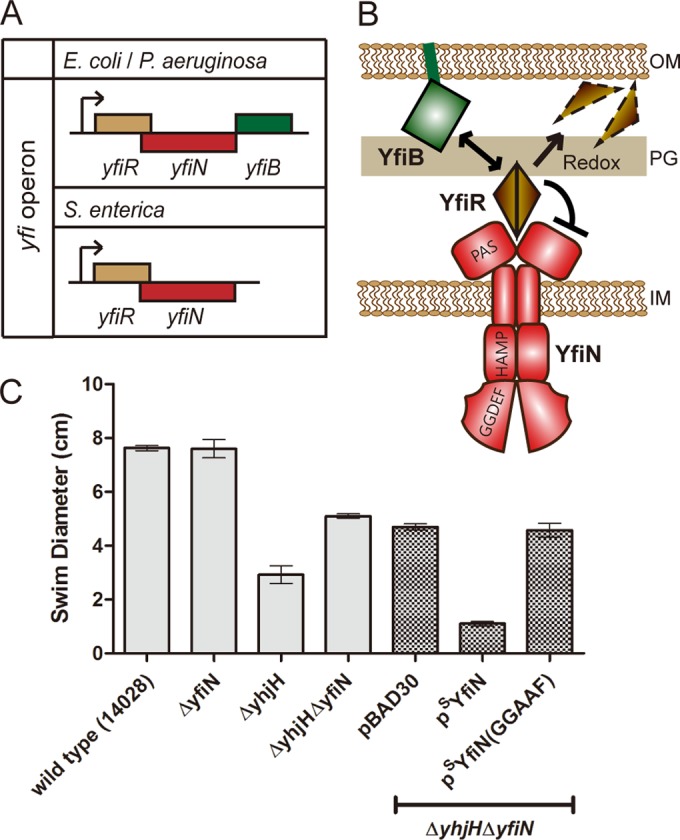
The c-di-GMP-synthetic enzyme YfiN contributes to intracellular c-di-GMP levels in *Salmonella*. (A) *yfi* operon organization in various bacteria. (B) Function of the Yfi system and YfiN domain organization as deduced from studies in *P. aeruginosa* (16, 19). (C) Swimming motility of *S. enterica* wild type (strain 14028) and indicated mutant derivative strains, some carrying an empty vector (pBAD30) or a vector expressing either ^S^YfiN or its active-site mutant ^S^YfiN (GGAAF). Overnight cultures of each strain were inoculated at the center of 0.3% agar swim plates supplemented with 0.2% arabinose and incubated at 37°C for 8 h. Error bars indicate standard deviations of the results from four experimental repeats.

The DGC YfiN, also called DgcN (14) or TpbB (15), is an inner membrane (IM) protein with a Per-Arnt-Sim (PAS)-like domain in the periplasm and histidine kinase, adenylate cyclase, methyl-accepting protein, and phosphatase (HAMP) and GGDEF domains in the cytoplasm. YfiN has been identified as a key contributor to intracellular c-di-GMP levels in various bacteria, and all of the YfiN-mediated cellular processes described to date are in keeping with the major role of c-di-GMP in inhibiting motility and promoting the biofilm state (15–18). In *Pseudomonas aeruginosa*, where the function of YfiN has been best studied (16, 19), YfiN was found to be regulated by YfiR and YfiB, which are encoded within the same operon (Fig. 1A). The periplasmic protein YfiR is proposed to inhibit YfiN, with the inhibition relieved by reducing conditions that misfold YfiR (19) or by the lipoprotein YfiB that sequesters YfiR to the outer membrane (OM) (Fig. 1B) (16). The *yfi* operon is widespread in Gram-negative bacteria but does not always include *yfiB* (19). For example, *yfiB* is absent in *Salmonella enterica* but present in *E. coli* and *P. aeruginosa* (Fig. 1A). Consistent with the proposal in *P. aeruginosa*, derepression of YfiN caused by disruption of the inhibitor YfiR enhances biofilm formation by activating cellulose production in *E. coli* (18, 20). In *Salmonella*, YfiN was reported to contribute to cellular c-di-GMP levels and inhibit motility through the c-di-GMP receptor YcgR (17). The Yfi system has been suggested to play an important role in the host colonization and persistence of *P. aeruginosa*, as well as a uropathogenic *E. coli* strain (16, 19, 20). We report here a novel second function for YfiN as an inhibitor of cell division in *E. coli* and *Salmonella*, a function promoted by the interaction of YfiN with components of the division machinery.

Bacterial cell division is orchestrated by the divisome, a dynamic multiprotein assembly that constricts cell envelope layers at the midcell, timed with completion of DNA replication (21–24). Cell division proteins assemble into the divisome broadly in two steps (23, 24). In an early step, well before the onset of cell constriction and while the cell is still elongating, the tubulinlike protein FtsZ forms a ring at the midcell, which is anchored to the membrane by two proteins, FtsA and ZipA (22, 23). Once assembled, this Z ring recruits downstream components to form a constriction-competent complex, which coordinates septum synthesis and invagination (cytokinesis) (23, 24). While FtsA and ZipA play redundant roles in anchoring the Z ring to the membrane, they are both essential for cytokinesis (22, 23, 25). FtsZ assembly is the major target of cell division checkpoints sensing various stresses, including DNA damage, defective cell wall synthesis, and nutrient starvation (26–29). Here, we show that in both *E. coli* and *Salmonella*, YfiN localizes to the midcell in a Z ring-dependent manner and halts cell division without disassembling the Z ring but, rather, by blocking its further progress toward cytokinesis. In *E. coli*, the midcell localization of YfiN, which requires FtsZ and ZipA, is stimulated by multiple conditions that cause cell envelope stress. Our data suggest that, while the primary role of the DGC YfiN is to promote biofilm formation under reducing conditions, it has a second role in inhibiting cell division in response to envelope stress.

## RESULTS

### YfiN contributes to intracellular c-di-GMP levels in *Salmonella* as measured by motility inhibition.

The initial impetus for this study was that, with the exception of the PDE YhjH, which was identified as a gatekeeper for maintaining low c-di-GMP levels in the cell and enabling motility (30, 31, 75), it was not known whether any of the other 18 GGDEF/EAL domain proteins in *Salmonella* were involved with motility regulation. Because YhjH would mask the contribution of these 18 proteins to cellular c-di-GMP levels, mutants with mutations in these genes were constructed in a Δ*yhjH* mutant, as well in a wild-type *Salmonella* background and examined for motility in a soft agar plate assay; the data are summarized in Fig. S1 in the supplemental material. Similar data were also recently published independently (17). Of the 18 proteins examined, four were seen to affect motility (see Fig. S1B). Of these, YfiN was observed to contribute most significantly (compare the results for the Δ*yhjH* mutant with those for the Δ*yhjH* Δ*yfiN* mutant in Fig. 1C; see also Fig. S1B). Overexpression of YfiN from an inducible plasmid (p^S^YfiN) resulted in impaired motility, while an active-site mutant of YfiN (GGDEF→GGAAF) failed to inhibit motility (Fig. 1C), results consistent with YfiN being a DGC.

Since the experiments described below study YfiN from three different bacteria, *E. coli*, *S. enterica*, and *P. aeruginosa*, we will henceforth use the superscripts E, S, and P, respectively, to indicate the bacterial source of YfiN or other proteins, as necessary. We will also use the subscripts green fluorescent protein (GFP), yellow fluorescent protein (YFP), and cyan fluorescent protein (CFP) for fluorescent fusion proteins, placed before or after the protein to indicate N- or C-terminal locations, respectively.

### YfiN accumulates at the midcell in a Z ring-dependent manner and negatively regulates cell division in *Salmonella*.

**FIG 2:**
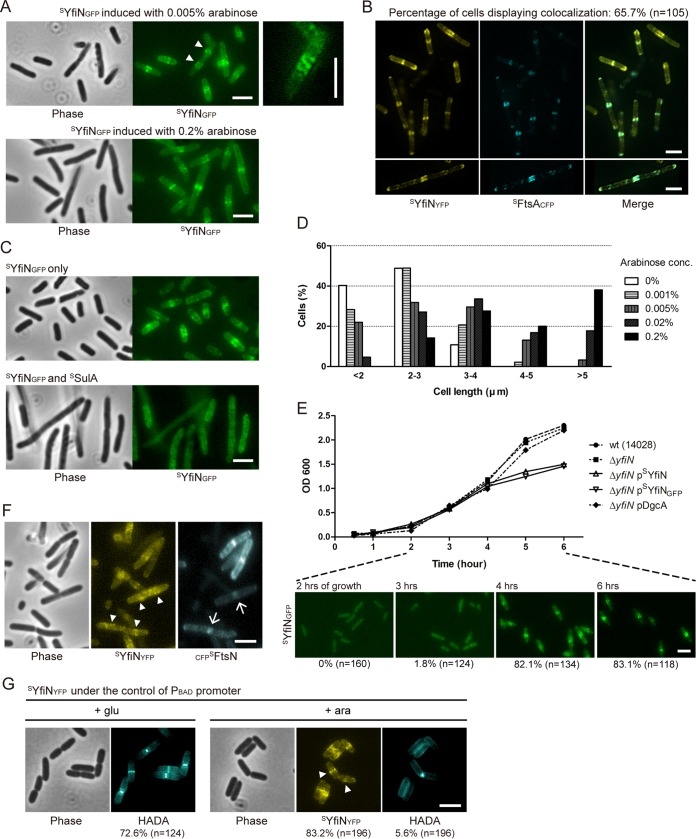
YfiN localizes to the division site, where it inhibits cell division in *Salmonella*. (A) YfiN localization at the midcell. Phase-contrast and fluorescence images of ^S^YfiN_GFP_ expressed from pBAD30 with different concentrations of inducer arabinose (0.005 and 0.2%) in a *Salmonella* Δ*yfiN* background. Cells with no visible constriction have a fluorescent YfiN band at the midcell, while cells with a constriction show YfiN at the quarter sites (arrowheads). The right panel zooms in on a representative cell showing a spiral structure of ^S^YfiN_GFP_ at the midcell. Unless otherwise noted, all strains in this study were grown with 0.005% arabinose at 30°C for 4 h before imaging. Scale bars, 3 µm in all images in this study. (B) Colocalization of YfiN and FtsA. ^S^YfiN_YFP_ and ^S^FtsA_CFP_ were coexpressed from pBAD plasmids in Δ*yfiN* cells. The bottom panels show a representative cell displaying colocalization of ^S^YfiN_YFP_ and ^S^FtsA_CFP_ in a spiral structure along the membrane. (C) Localization of YfiN in the absence and presence of SulA. After 3 h of growth, the expression of ^S^YfiN_GFP_ and ^S^SulA from pBAD plasmids was induced in a Δ*yfiN* strain, and cells were grown for one further hour before imaging. (D) Histogram of cell length distribution of Δ*yfiN* cells expressing ^S^YfiN with different concentrations of the inducer arabinose. Cell length was measured manually as the distance between two poles, and the distribution is shown as percentages of cells in the indicated ranges. The cell numbers in different concentrations of arabinose were as follows: 0%, *n* = 154; 0.001%, *n* = 192; 0.005%, *n* = 191; 0.02%, *n* = 197; and 0.2%, *n* = 210. (E) Growth curves of the wild type (strain 14028) and its Δ*yfiN* and Δ*yfiN* derivatives expressing ^S^YfiN, ^S^YfiN_GFP_, or DgcA from pBAD30. The inducer arabinose (0.005%) was added at time zero in this experiment, and the localization of ^S^YfiN_GFP_ at selected time points is shown below. The percentages of cells showing ^S^YfiN_GFP_ at the midcell and the total number of cells counted are shown below the images. (F) Localization of YfiN and FtsN. ^S^YfiN_YFP_ and _CFP_^S^FtsN were coexpressed from pBAD plasmids in Δ*yfiN* cells. Arrowheads denote localization of ^S^YfiN to the quarter positions, and arrows indicate ^S^FtsN foci localizing at midcell constriction sites. (G) Nascent peptidoglycan synthesis in cells expressing YfiN. Δ*yfiN* cells expressing ^S^YfiN_YFP_ from pBAD33 were grown with either glucose or arabinose and labeled with the blue-fluorescent d-amino acid HADA for 1 min. The percentages of cells displaying HADA or ^S^YfiN_YFP_ at the midcell and the total number of cells counted are shown below the images.

Several GGDEF/EAL proteins have been reported to have a distinct subcellular localization or to exist in a complex with their downstream targets (7, 11, 32–34), prompting us to examine the localization of YfiN. A fusion of GFP to *Salmonella* YfiN (^S^YfiN_GFP_) was expressed from an inducible plasmid and confirmed to be functional (see Fig. S2A in the supplemental material). In a Δ*yfiN* background, ^S^YfiN_GFP_ showed as a fluorescent band at the midcell (Fig. 2A), suggesting an association with the cell division machinery. Some of the cells displayed spiral-like structures (Fig. 2A, right), implying that YfiN may associate with the Z ring, whose intermediate structures in various bacteria appear in a spiral/helical configuration at the midcell (35–37). The cytoskeleton protein MreB has also been proposed to polymerize into helical structures in the cell (38), but ^S^YfiN_GFP_ maintained its structures in the presence of A22, an inhibitor of MreB polymerization (see Fig. S3A). Interestingly, when ^S^YfiN_GFP_ was at the midcell, no visible cell constriction could be observed (Fig. 2A), whereas in cells with a constriction, ^S^YfiN_GFP_ was at the quarter positions, which are future division sites (Fig. 2A, arrowheads). These observations indicate that YfiN is likely recruited to the midcell by early division proteins, such as FtsZ, FtsA, and ZipA, prior to constriction.

To detect colocalization of ^S^YfiN with FtsZ, we used FtsA, the essential division protein that anchors FtsZ to the membrane (22), as a proxy, because cells expressing FtsZ fluorescent fusions grew poorly. In cells coexpressing ^S^YfiN_YFP_ and ^S^FtsA_CFP_, the two proteins colocalized within rings at the midcell in a majority of the cells (Fig. 2B), as well as within spiral structures along the length of the cell (Fig. 2B, bottom). When the Z ring was disassembled by the expression of the SOS cell division inhibitor SulA (39), ^S^YfiN_GFP_ failed to localize to the midcell (Fig. 2C). These results suggest that the recruitment of YfiN to the division site is dependent on the assembly of the Z ring.

The accumulation of ^S^YfiN at the division site was accompanied by cell lengthening in an inducer (arabinose) concentration-dependent manner (Fig. 2A): at 0.2% inducer concentration, cells were approximately twice as long as without inducer (Fig. 2D), indicating that cell division is blocked by YfiN. In addition, cells expressing either ^S^YfiN or ^S^YfiN_GFP_ showed a growth defect concomitant with the midcell accumulation of ^S^YfiN_GFP_ (Fig. 2E). No cell lengthening or growth defect was observed in cells overexpressing a constitutively active DGC, DgcA, from *Caulobacter crescentus* (Fig. 2E) (40), whose activity was confirmed in motility assays (see Fig. S2B in the supplemental material), indicating that the cell division defect caused by YfiN is not merely a consequence of elevated c-di-GMP levels.

The absence of a visible midcell invagination and only a moderate cell lengthening in cells expressing ^S^YfiN suggests that YfiN inhibits Z ring constriction, as well as septal peptidoglycan (PG) synthesis. Constriction begins after the last essential division protein, FtsN, is recruited to the midcell; the arrival of FtsN has been suggested to activate septal PG synthesis (41, 42). To determine whether YfiN prevents the recruitment of FtsN, we examined the localization of _CFP_^S^FtsN in cells expressing ^S^YfiN_YFP_. The majority of cells (79.5%; *n* = 239) expressing both proteins showed the presence of either one or the other protein, but not both, at the midcell (Fig. 2F). While no cell showed colocalization of ^S^YfiN_YFP_ and _CFP_^S^FtsN at the midcell, some cells that showed a visible septal invagination (10.4%) exhibited distinct localization of the two fluorescent proteins, with ^S^YfiN_YFP_ at the quarter positions (Fig. 2F, arrowheads) and _CFP_^S^FtsN at the constricting septum (arrows).

To examine the effects of YfiN on PG synthesis, we made use of a fluorescent d-amino acid that labels sites of nascent PG synthesis through incorporation into the cell wall (43). In the majority of cells in which the expression of ^S^YfiN_YFP_ was repressed by glucose, the blue-fluorescent d-amino acid HADA was found incorporated as a band at the midcell, regardless of whether a cell constriction was visible (Fig. 2G). In contrast, when the expression of ^S^YfiN_YFP_ was induced by arabinose, the number of cells showing HADA at the midcell decreased from 72.6% to 5.6% (Fig. 2G, compare + glu to + ara), which indicates that YfiN inhibits septal PG synthesis. In the 5.6% of cells that showed HADA at the constriction site, ^S^YfiN_YFP_ was exclusively at the quarter sites (Fig. 2G, arrowheads). The stalled septal PG synthesis and the distinct localization patterns of YfiN and FtsN raise the possibility that YfiN accumulation prevents the Z ring from maturing into a constriction-competent division complex, possibly by inhibiting the recruitment of late division proteins.

### YfiN is recruited to the division site in response to cell envelope stress in *E. coli*.

**FIG 3:**
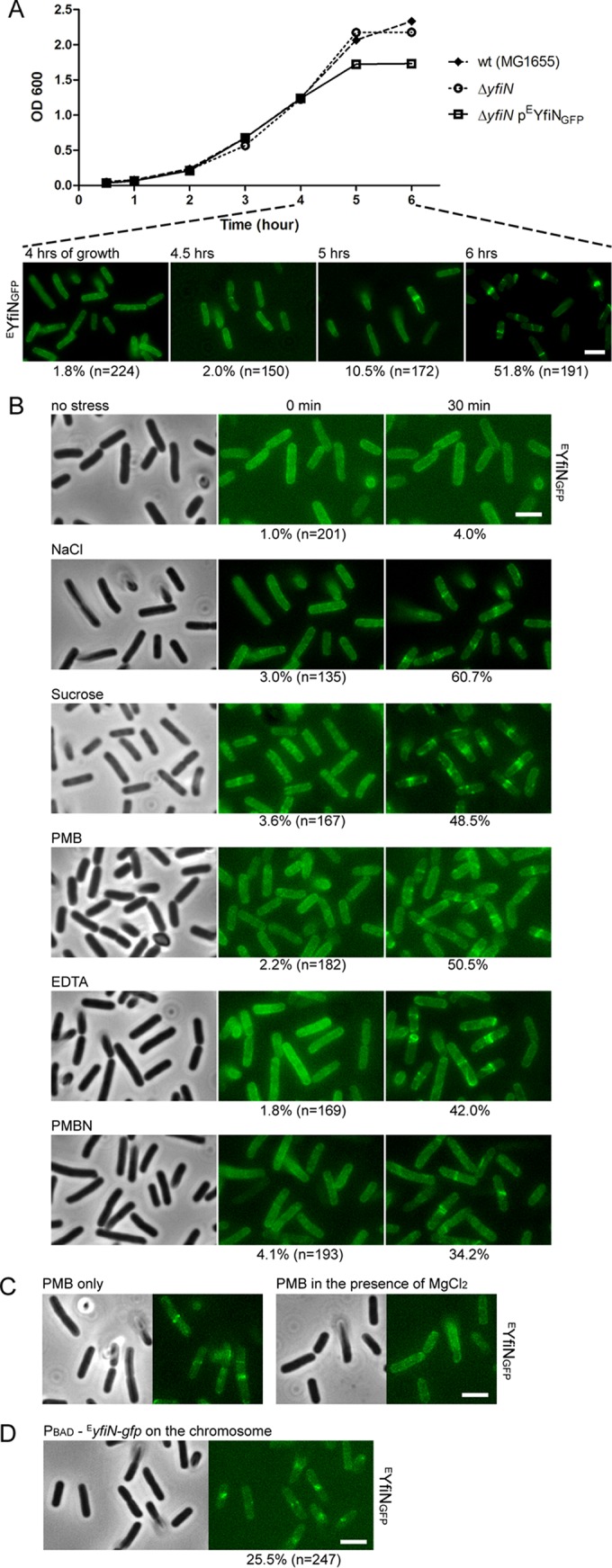
YfiN relocates to the midcell in response to envelope stress in *E. coli*. (A) Growth curves of *E. coli* wild-type (strain MG1655) cells, Δ*yfiN* cells, or Δ*yfiN* cells expressing ^E^YfiN_GFP_ from pBAD30 with the inducer arabinose added at time zero. The localization of ^E^YfiN_GFP_ at selected time points is shown in the images below. (B) ^E^YfiN relocation to the midcell upon envelope stress. *E. coli* Δ*yfiN* cells expressing ^E^YfiN_GFP_ were exposed to the indicated stresses. The same microscope fields were photographed before and 30 min after exposure to no stress (LB medium only), osmotic upshift (LB with 250 mM additional NaCl or 10% sucrose), or OM permeabilization (LB with 2.5 µg/ml PMB, 10 mM EDTA, or 200 µg/ml PMBN). For each stressor, the same field of cells was observed to count the fraction of cells showing midcell foci. (C) Effect of added Mg^2+^ on PMB-triggered ^E^YfiN relocation. *E. coli* Δ*yfiN* cells expressing ^E^YfiN_GFP_ were exposed to 2.5 µg/ml PMB for 30 min in the absence and presence of 10 mM MgCl_2_. (D) Localization of ^E^YfiN_GFP_ expressed from the chromosomal inducible promoter. An *E. coli yfiR::kan* mutant strain, in which *^E^*yfiN-gfp is carried under the P_BAD_ promoter on the chromosome, was grown with arabinose at 30°C for 4 h and exposed to 250 mM NaCl for 30 min.

To determine whether YfiN has the same cell division arrest function in the closely related bacterium *E. coli*, a GFP fusion of *E. coli* YfiN (^E^YfiN_GFP_) was constructed and confirmed to be functional (see Fig. S2B in the supplemental material). Analysis of intracellular c-di-GMP levels in cells expressing ^E^YfiN_GFP_ was consistent with the designation of ^E^YfiN as a DGC (see Materials and Methods). When expressed in an *E. coli* Δ*yfiN* background, ^E^YfiN_GFP_ localized to the midcell, concomitant with a growth defect (Fig. 3A), similar to the experiment whose results are shown in Fig. 2E. A membrane-dispersed localization of ^E^YfiN_GFP_ was clearly evident until the mid-log phase (after 4 h of growth), and one or more hours of further growth was required for ^E^YfiN_GFP_ to relocate to the midcell (Fig. 3A).

The relocation of ^E^YfiN_GFP_ to the midcell near the stationary phase of growth suggested that the midcell localization might be a response to depletion of nutrients, changes in pH, or some other stressful condition. Previous studies in *P. aeruginosa* and *E. coli* have shown that misfolding of the periplasmic inhibitor YfiR caused by reducing environments leads to activation of YfiN, identifying reductive stress as one of the input signals of the Yfi system (18, 19). Additionally, in *P. aeruginosa*, the Yfi system was proposed to contribute to biofilm formation under cell envelope stress conditions like osmotic upshift and exposure to the detergent sodium dodecyl sulfate (SDS) (19). To identify input stimuli that promote the observed ^E^YfiN relocation to the midcell, we tested conditions suggested to activate the Yfi system in previous studies and a variety of other stressors as well, including nutrient starvation and acid stress. Cells producing ^E^YfiN_GFP_ were exposed to a stress condition after 4 h of growth, when ^E^YfiN_GFP_ was still dispersed throughout the membrane (Fig. 3A). Of the many stressors tested, only the following conditions were observed to trigger ^E^YfiN_GFP_ relocation within 30 min of exposure at room temperature: osmotic upshift with either NaCl (250 mM) or sucrose (10%) and treatment with the envelope-targeting antibiotic polymyxin B (PMB; 2.5 µg/ml) (Fig. 3B). These treatments stimulated ^E^YfiN relocation in a concentration-dependent manner (see Fig. S4A and B in the supplemental material); osmotic downshift had no impact. No new protein synthesis was required for this response (see Fig. S5).

PMB, a polycationic molecule, is thought to increase cell permeability in Gram-negative bacteria by interacting with both the outer membrane (OM) and inner membrane (IM) in a dual mechanism of action (44–46). PMB first binds to the negative charges on lipopolysaccharide (LPS) and removes the divalent cations that stabilize the LPS structure (44, 45). This results in an increase in OM permeability, which allows PMB to penetrate into the IM, causing leakage of cell contents and cell death (44, 45). To determine whether alteration of the OM or the IM stimulates the ^E^YfiN relocation, we tested two more agents, EDTA and polymyxin B nonapeptide (PMBN). EDTA is a strong divalent cation chelator known to disrupt the OM in the same manner as PMB, but EDTA is not an IM stressor (44, 46). The polymyxin derivative PMBN is less lethal than PMB due to the absence of the fatty acid tail required for the IM disruption, but PMBN still retains the ability to permeabilize the OM (44). Both of the OM-permeabilizing agents, EDTA (10 mM) and PMBN (200 µg/ml), induced midcell localization of ^E^YfiN_GFP_ (Fig. 3B), indicating that OM disruption is most likely the trigger for ^E^YfiN relocation. In agreement with the known property of high divalent cation concentrations in blocking the effect of PMB on OM permeabilization (44), the addition of external MgCl_2_ (10 mM) prevented PMB treatment from triggering ^E^YfiN_GFP_ relocation (Fig. 3C). Other membrane-targeting agents, including a β-lactam antibiotic, SDS, and lysozyme, failed to relocate ^E^YfiN_GFP_ (see Fig. S4C in the supplemental material). Taken together, these data suggest that envelope stress caused by osmotic upshift or OM permeabilization by divalent ion extraction is the input signal for ^E^YfiN to relocate to the division site.

While ^E^YfiN_GFP_ relocated to the midcell in response to envelope stress, the same conditions had no effect on ^S^YfiN_GFP_ localization in *Salmonella* (not shown). This observation led us to hypothesize that the lipoprotein YfiB, which is absent in *Salmonella*, might function as a sensor that couples envelope stress to ^E^YfiN localization (Fig. 1A and B). It is worth noting that YfiB is a structural homolog of Pal, a component of the Tol-Pal complex that plays a crucial role in maintaining OM integrity in Gram-negative bacteria (47, 48). However, in a *yfiB* knockout mutant of *E. coli*, ^E^YfiN_GFP_ still retained its ability to relocate upon envelope stress (not shown), indicating that YfiB is not involved in the ^E^YfiN relocation.

Next, we fused GFP to the *yfiN* chromosomal locus in *E. coli* to observe the localization of endogenously expressed ^E^YfiN. However, ^E^YfiN_GFP_ expressed under its native promoter failed to display enough fluorescence to be observed regardless of exposure to stress (not shown). When the native promoter was replaced with an inducible promoter (P_BAD_), ^E^YfiN_GFP_ expressed from the chromosomal locus was able to show relocation upon the envelope stress (Fig. 3D). These data suggest that a single copy on the chromosome is sufficient to produce ^E^YfiN serving as a cell division inhibitor, but it requires activation at the transcriptional level by as-yet-unknown signals.

### Localization of ^E^YfiN to the division site is dependent on its interaction with FtsZ and ZipA.

Having observed that ^S^YfiN is associated with the Z ring (Fig. 2B and C), we monitored ^E^YfiN_GFP_ localization in temperature-sensitive mutants of *E. coli* cell division proteins in order to validate specific binding targets of ^E^YfiN. We used *ftsZ84* (strain WM1125), *ftsA12* (strain WM1115), and *zipA1* (strain PS223) mutant strains that are defective in the assembly/recruitment of the respective protein to the division site at the nonpermissive temperature (42°C). The essential division proteins FtsA and ZipA serve as redundant membrane anchors for the Z ring (22, 25). At the nonpermissive temperature (42°C), lack of Z ring assembly in the *ftsZ84* mutant results in loss of localization of all divisome components (49). The absence of either FtsA or ZipA in the *ftsA12* or *zipA1* mutant at 42°C is not expected to disrupt the Z ring because FtsZ remains at the midcell as long as either one of these proteins is present, but assembly of downstream components that mediate cytokinesis is inhibited in both of these mutants (25, 49).

**FIG 4:**
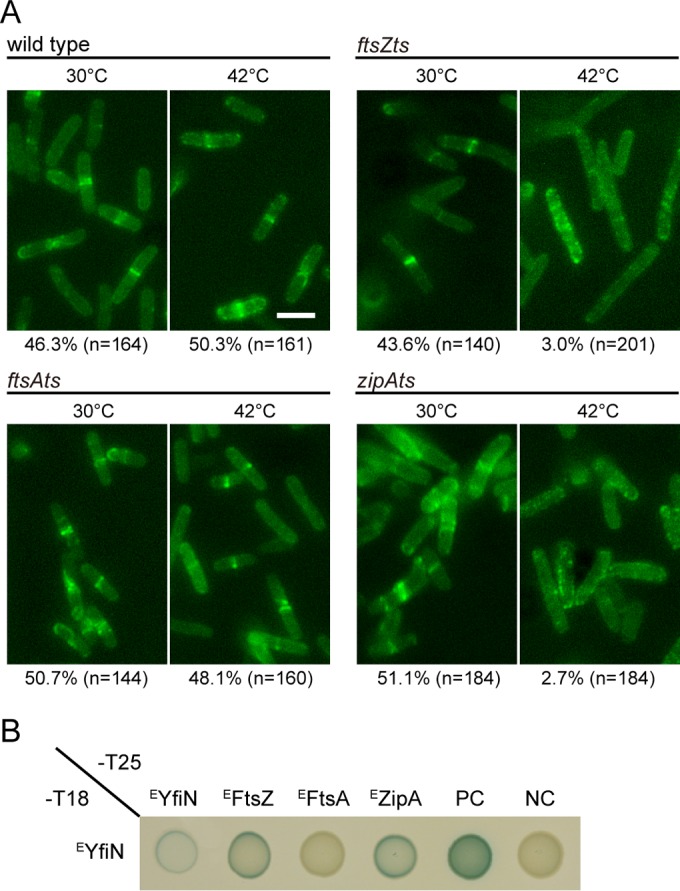
YfiN interacts with FtsZ and ZipA in *E. coli*. (A) Localization of ^E^YfiN_GFP_ in wild-type (MG1655) cells and temperature-sensitive (ts) mutants with mutations of cell division proteins in *E. coli*. For each strain, cells producing ^E^YfiN_GFP_ were exposed to 2.5 µg/ml PMB for 30 min. Following the exposure, cells were incubated for another 30 min at two different temperatures, 30°C and 42°C, before imaging. (B) Bacterial adenylate cyclase two-hybrid (BACTH) analysis of *E. coli* division proteins against ^E^YfiN. Either the T18 or T25 fragment was fused to the C-terminal ends of ^E^YfiN and the cell division proteins. PC, positive control (T18-leucine zipper/T25-leucine zipper); NC, negative control (T18/T25 empty vectors).

The *E. coli* mutant cells expressing ^E^YfiN_GFP_ were first exposed to PMB for 30 min at room temperature to promote ^E^YfiN relocation and then shifted to 30°C or 42°C for another 30 min. At 30°C, ^E^YfiN_GFP_ remained in spiral/ring structures at the midcell in a wild-type background and in all three mutants (Fig. 4A). At 42°C, while wild-type cells maintained ^E^YfiN_GFP_ at the midcell, both the *ftsZ84* and the *zipA1* mutant lost the localization and showed dispersed clusters of ^E^YfiN_GFP_ (Fig. 4A), suggesting that ^E^FtsZ and ^E^ZipA are essential for ^E^YfiN localization. In contrast, ^E^YfiN_GFP_ localization was unaffected in the *ftsA12* mutant at 42°C (Fig. 4A), indicating that ^E^YfiN_GFP_ localization to the midcell does not require ^E^FtsA and, therefore, also does not require cell division components that act downstream from ^E^FtsA. NaCl was not used as a stressor in these experiments because the *ftsZ84* mutation can be suppressed by high salt (50). However, similar results were obtained when EDTA was used as a stressor (see Fig. S6 in the supplemental material). In summary, these data show that ^E^YfiN localization at the midcell depends on the ^E^FtsZ ring and its ^E^ZipA tether but not on ^E^FtsA or downstream events known to be dependent on ^E^FtsA assembly.

To further investigate interactions between ^E^YfiN and the cell division proteins, the bacterial adenylate cyclase two-hybrid (BACTH) assay was performed. The BACTH assay has been used successfully for analyzing interactions between membrane proteins, including cell division and cytoskeleton proteins (51, 52). For this assay, FtsZ, FtsA, ZipA, and YfiN from *E. coli* were fused to two fragments of the *Bordetella pertussis* adenylate cyclase, T18 and T25, and their interaction was monitored by measuring the synthesis of β-galactosidase, which is dependent on the adenylate cyclase activity. The results showed self-interaction of ^E^YfiN (Fig. 4B), which is expected because dimerization is required for DGCs to exert their enzymatic activity (53). A positive result was obtained with ^E^YfiN and either ^E^FtsZ or ^E^ZipA, supporting their interaction (Fig. 4B). No interaction was detected between ^E^YfiN and ^E^FtsA (Fig. 4B), in agreement with the persistence of ^E^YfiN_GFP_ at the midcell in the *ftsA12* mutant at 42°C (Fig. 4A). The cytoskeletal protein ^E^MreB, previously shown to colocalize with ^E^FtsZ and directly interact with it using the BACTH assay (52), showed interaction with ^E^FtsZ but not with ^E^YfiN (see Fig. S3B in the supplemental material). Taken together, the data in Fig. 4 and in Fig. S3 in the supplemental material show that ^E^YfiN relocation at the midcell is dependent on ^E^FtsZ and ^E^ZipA but not on ^E^FtsA or ^E^MreB.

### High intracellular c-di-GMP levels are required for midcell localization of YfiN in *E. coli*.

**FIG 5:**
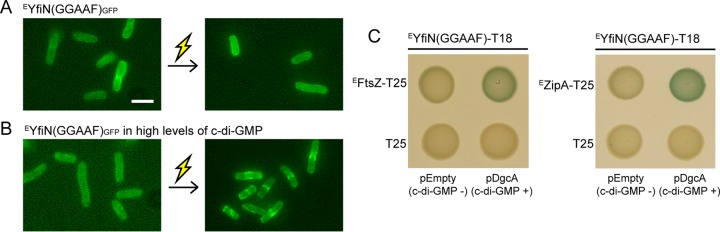
High levels of c-di-GMP provided by a heterologous DGC restore midcell relocation of an active-site mutant of *E. coli* YfiN. (A and B) *E. coli* Δ*yfiN* cells expressing ^E^YfiN(GGAAF)_GFP_ alone (A) or with the *C. crescentus* DGC DgcA (B) were grown with inducer [arabinose for ^E^YfiN(GGAAF)_GFP_ and 0.01 mM IPTG for DgcA] at 30°C for 4 h and exposed to 250 mM NaCl. Images were taken before and 30 min after the osmotic upshift. Thunderbolt represents envelope stress. (C) BACTH analysis of c-di-GMP-stimulated interaction between ^E^YfiN(GGAAF) and cell division proteins ^E^FtsZ and ^E^ZipA. Along with the T18 and T25 constructs, each strain contains an empty pBAD33 or a plasmid carrying *dgcA*, whose expression was induced with 0.2% arabinose. Overexpression of DgcA alone did not affect the results of BACTH, as shown with control strains in the bottom row.

Given the unexpected role of the c-di-GMP-synthetic enzyme YfiN in cell division regulation, we wondered whether the DGC activity of YfiN was required for its cell division arrest function or whether this was a separate function of YfiN. We therefore inactivated the DGC active site of ^E^YfiN_GFP_ (GGDEF→GGAAF). Like the wild-type protein, the mutant protein was evenly distributed throughout the membrane before exposure to stress but failed to localize to the midcell when exposed to osmotic upshift (Fig. 5A), suggesting that relocation of YfiN to the division site is dependent on the DGC activity. To determine whether the requirement for the DGC activity can be bypassed by high levels of c-di-GMP, we provided c-di-GMP artificially by expressing the constitutively active heterologous DGC DgcA (40). Under this condition, the mutant ^E^YfiN was able to relocate to the midcell in response to envelope stress (Fig. 5B). This suggests that high intracellular c-di-GMP levels are required for ^E^YfiN to interact with cell division proteins. To assess the c-di-GMP dependence of YfiN interaction with division proteins, BACTH analysis was performed again. The interaction of the mutant ^E^YfiN with ^E^FtsZ or ^E^ZipA was observed to be strengthened in the presence of DgcA (Fig. 5C), consistent with the localization data (Fig. 5B). These results suggest two possible models: (i) c-di-GMP directly binds to ^E^YfiN, which then causes its direct interaction with cell division proteins, and (ii) c-di-GMP indirectly promotes ^E^YfiN interaction with cell division proteins via some other factor that binds c-di-GMP.

### Midcell relocation of YfiN in response to envelope stress requires release of its periplasmic inhibitor YfiR by reductive stress in *E. coli*.

**FIG 6:**
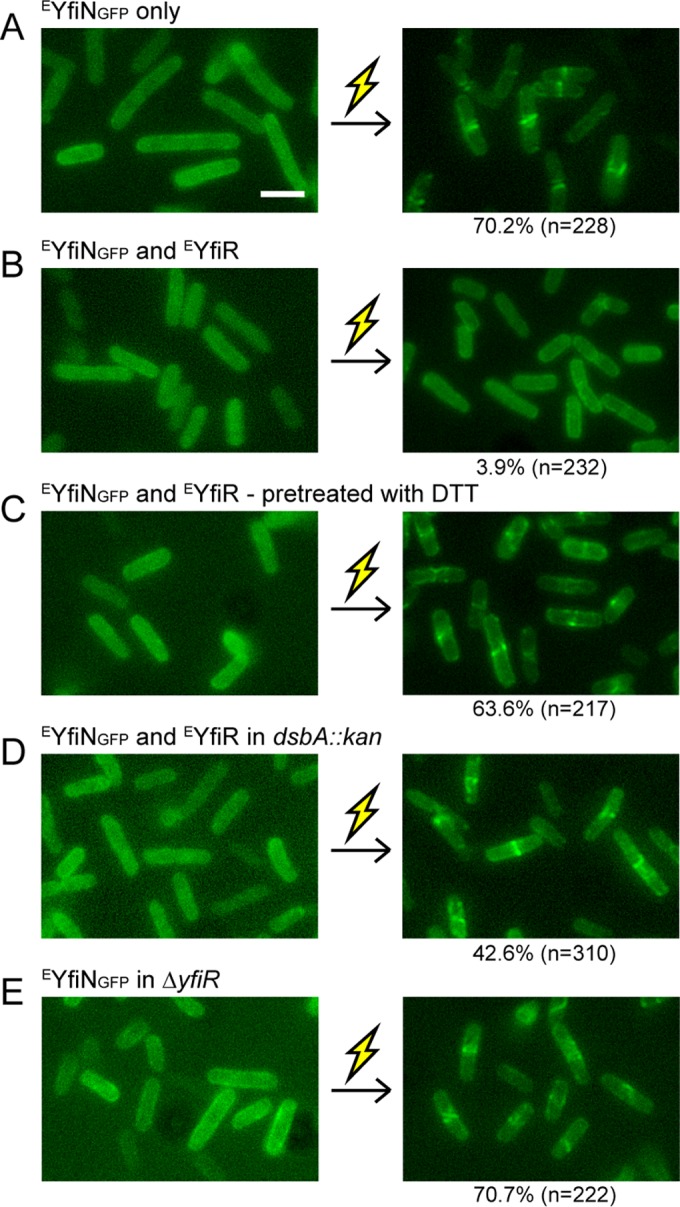
YfiR prevents YfiN relocation to the midcell in *E. coli*. Cells were grown with inducer (arabinose for ^E^YfiN_GFP_ and 0.01 mM IPTG for ^E^YfiR) at 30°C for 4 h and exposed to 250 mM NaCl. Images were taken before and 30 min after the osmotic upshift. Thunderbolt represents envelope stress. (A) *E. coli* Δ*yfiN* cells expressing ^E^YfiN_GFP_ only. (B) *E. coli* Δ*yfiN* cells expressing ^E^YfiN_GFP_ and ^E^YfiR. (C) *E. coli* Δ*yfiN* cells expressing ^E^YfiN_GFP_ and ^E^YfiR. Ten millimoles DTT was added to the culture 1 h prior to osmotic upshift. (D) *E. coli dsbA::kan* cells expressing ^E^YfiN_GFP_ and ^E^YfiR. (E) *E. coli* Δ*yfiR* cells expressing ^E^YfiN_GFP_ only.

The periplasmic protein YfiR inhibits the c-di-GMP-synthetic activity of YfiN by interacting with the PAS-like domain of YfiN in the periplasm (16, 18, 20). To assess whether YfiR also counteracts the function of YfiN in cell division, ^E^YfiR was coexpressed with ^E^YfiN_GFP_ in *E. coli*. In the presence of ^E^YfiR, ^E^YfiN_GFP_ remained localized throughout the membrane and failed to relocate to the midcell when cells were exposed to envelope stress (compare Fig. 6A and B). These results indicate that ^E^YfiR is a repressor of the cell division arrest function of ^E^YfiN as well.

Previous studies in *P. aeruginosa* and *E. coli* suggested that YfiR is a periplasmic redox sensor that regulates YfiN activity in response to reducing conditions (18, 19). YfiR has two pairs of conserved cysteine residues whose intramolecular disulfide bonds play important roles in dimerization (54). Reducing environments are thought to disrupt the disulfide bonds in YfiR, thus derepressing YfiN (18, 19, 54). To test whether the inhibitory effect of ^E^YfiR on ^E^YfiN relocation can be relieved by reducing conditions, *E. coli* cells coexpressing ^E^YfiN_GFP_ and ^E^YfiR were treated with the reducing agent dithiothreitol (DTT). When DTT was added to a final concentration of 10 mM for 1 h prior to envelope stress exposure, ^E^YfiR lost its ability to repress ^E^YfiN relocation (Fig. 6C). Similar results were obtained in the absence of DsbA, a protein responsible for disulfide bond formation in periplasmic proteins (Fig. 6D). The inactivation of the disulfide bonding system (DSB) has been reported to relieve the repression of YfiN by YfiR (18, 19). Under both reducing conditions (Fig. 6C and FigD), ^E^YfiN remained dispersed in the membrane until exposed to envelope stress, which indicates that the release of ^E^YfiR is required but not sufficient for the relocation of ^E^YfiN. These results were also supported by the results in a *yfiR* knockout mutant (Fig. 6E).

Overall, these data identify ^E^YfiN as a sensor that detects two different extracellular signals. A reductive stress signal is required to inactivate the inhibitor ^E^YfiR and turn on the DGC activity of ^E^YfiN, which is essential for responding to envelope stress and relocating YfiN to the midcell (Fig. 5). Thus, ^E^YfiN senses and responds to two sequential signals—reductive and envelope stresses—before arresting cell division.

### YfiN upregulation offers protection against polymyxin B in *E. coli*.

**FIG 7:**
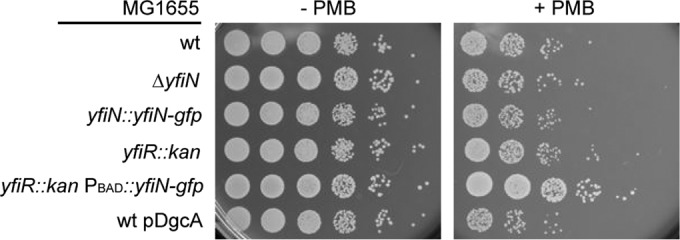
Ectopically expressed ^E^YfiN enhances cell viability after exposure to polymyxin B. After growth to the mid-log phase (at 30°C for 4 h) with 0.005% arabinose, the indicated strains were incubated with or without 2.5 µg/ml PMB for 30 min at room temperature and plated in 10-fold dilutions (10^−1^ to 10^−6^) on LB agar plates. In the *yfiR*::k*an* mutants, the *kan* cassette is inserted in an orientation opposite to the direction of *yfi* operon transcription in order to avoid polar effects on the downstream *yfiN* gene.

The dynamic relocation of ^E^YfiN to the division site in response to potentially lethal envelope stressors suggests that this response might provide protection and/or facilitate adaptation under such conditions. To test this, we examined the following several strains for their susceptibility to PMB: wild-type *E. coli* strain MG1655, the Δ*yfiN* mutant, a strain carrying *yfiN-gfp* at its native chromosomal locus, and Δ*yfiR* and Δ*yfiR* mutants with ^E^YfiN_GFP_ expressed from the chromosomal inducible promoter (also used in the experiment whose results are shown in Fig. 3D). Of these strains, only the one with ectopic expression of ^E^YfiN from the chromosomal inducible promoter showed an approximately 10-fold increase in survival after 30 min of exposure to PMB (2.5 µg/ml) (Fig. 7). While these data do not address whether the division arrest function of ^E^YfiN is the cause of increased cell survival, they implicate ^E^YfiN in participating in bacterial defense mechanisms against envelope stress. There was no difference in survival between the wild type and the Δ*yfiN* strain, indicating that the ^E^*yfiN* gene under the control of its native promoter needs to be upregulated by unknown signals in order to offer protection. The data also show that the protection against PMB is not simply a consequence of elevated c-di-GMP levels, because overexpression of DgcA did not increase survival (Fig. 7).

## DISCUSSION

Since the original discovery of c-di-GMP as an allosteric effector of a bacterial cellulose synthase (55) and the later revelation of its major role as a second messenger that controls the decision between motile and sedentary bacterial lifestyles, the function of c-di-GMP has steadily expanded to include a remarkably diverse set of cellular processes. In this work, we establish a new and unique role for the diguanylate cyclase YfiN as both an enzyme and an effector that stalls cell division by interacting with early cell division proteins in *E. coli* and *Salmonella*.

### YfiN as a sensor for multiple environmental stresses.

In both *Salmonella* and *E. coli*, YfiN localizes to the division site and arrests cell division, which is exerted through interaction with cell division proteins (Fig. 2, 3, and 4). In *E. coli*, the trigger for recruitment of YfiN to the midcell is osmotic upshift and membrane permeabilization (Fig. 3B). The OM permeabilizers that stimulate ^E^YfiN relocation—PMB, EDTA, and a high concentration of PMBN (Fig. 3B)—have all been reported to induce the release of LPS and leakage of periplasmic proteins by altering LPS-LPS interactions in the OM (44, 56). Hyperosmotic stress also causes periplasmic contents to leak out (44, 57). This common attribute of the agents suggests that periplasmic leakage might be the specific input signal for the ^E^YfiN-mediated cell division inhibition. One might then imagine that loss of the periplasmic protein YfiR by periplasmic leakage might be the trigger of ^E^YfiN relocation, given the function of YfiR as an inhibitor of YfiN (16, 18). However, this is not the case, because inactivation of ^E^YfiR by either reductive stress or genetic mutation was not enough to relocate ^E^YfiN to the midcell without envelope stress (Fig. 6C, D, and E). Thus, ^E^YfiN is a membrane-associated sensor that responds to two independent environmental cues, reductive and envelope stresses.

While midcell localization of YfiN in *E. coli* is stimulated by osmotic upshift and OM permeabilization, the same conditions did not lead to a change in YfiN localization in *Salmonella* (not shown), suggesting that the signals that cause division arrest by YfiN differ between *E. coli* and *Salmonella*. In both bacteria, YfiN midcell localization was dependent on the growth phase (Fig. 2E and 3A), but the exact stress sensed in the stationary phase is not known. Surprisingly, *P. aeruginosa* YfiN does not localize to the division site either during the stationary phase or with envelope stress exposure in any of the bacterial species tested (see Fig. S7 in the supplemental material). Thus, despite its overall conservation as a c-di-GMP-synthetic enzyme, YfiN appears to have evolved to acquire an additional cell division arrest function in *E. coli* and *Salmonella*, which is activated in response to different environmental signals*.*

Thus far, DGCs are known to produce c-di-GMP in response to environmental cues, and c-di-GMP then serves as a second messenger that binds to a protein or an RNA effector and elicits a downstream response. A DGC-inactive ^E^YfiN regained the ability to interact with cell division proteins when intracellular c-di-GMP levels were elevated (Fig. 5B and C). This suggests that ^E^YfiN in a c-di-GMP-bound state itself might be the effector that interacts with the division proteins. Alternatively, c-di-GMP could bind to some other division protein that in turn promotes the recruitment of ^E^YfiN to the division site. However, the latter possibility is less likely because, while the c-di-GMP-binding proteins known to date include many GGDEF domain proteins, they do not include those constituting the divisome machinery (58). Although ^E^YfiN does not have a conserved I site (RXXD motif), which is the best characterized c-di-GMP-binding site in GGDEF domain proteins, a recent study reported an example of a DGC that exists in a complex with c-di-GMP even when it has no I site (59). We therefore favor the idea that high levels of c-di-GMP might enhance the interaction of ^E^YfiN with division proteins by binding to ^E^YfiN and stabilizing the conformational change induced by envelope stress.

**FIG 8:**
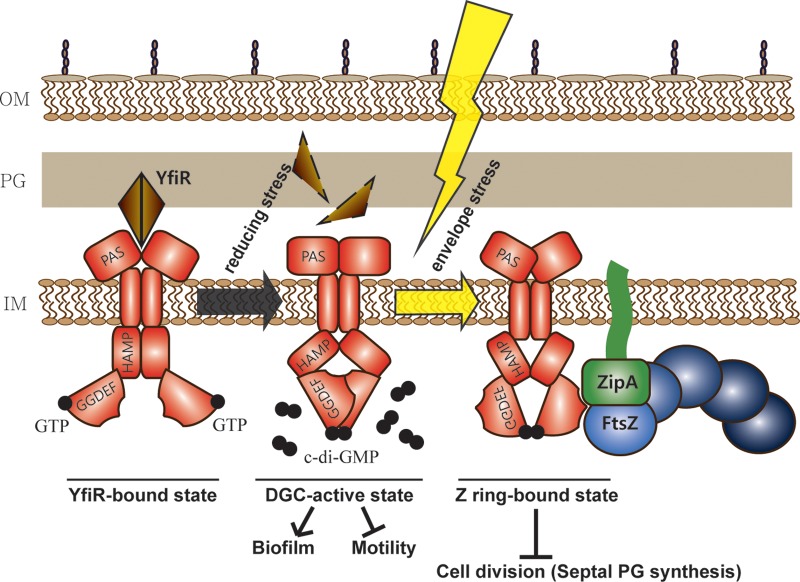
Model for YfiN as a cell division inhibitor. ^E^YfiN is a bifunctional protein that responds to two different environmental stresses—reductive and envelope stress. The DGC function is known to be activated when reductive stress inactivates the periplasmic repressor ^E^YfiR. The c-di-GMP thus produced inhibits motility and activates biofilm formation. The second function of ^E^YfiN as a cell division inhibitor, as revealed in this study, requires an additional envelope stress after the DGC function is activated. The envelope stress-induced conformation of ^E^YfiN, which is likely in a c-di-GMP-bound state, exposes binding sites for division proteins FtsZ and ZipA, directing ^E^YfiN to the future division site, where it halts division by preventing the initiation of septal peptidoglycan synthesis.

Based on these results, we propose a model in which ^E^YfiN acts as a bifunctional protein exhibiting both enzymatic and effector activities (Fig. 8). The enzymatic function of ^E^YfiN is activated by the release of ^E^YfiR under reducing conditions, and the c-di-GMP thus generated impairs motility and enhances biofilm formation (18, 20). The effector function exerted by c-di-GMP-bound ^E^YfiN requires an additional envelope stress cue, which likely results in an additional conformation change that exposes binding sites on ^E^YfiN to the division proteins FtsZ and ZipA, leading to cell division arrest.

The idea of bifunctional GGDEF/EAL domain proteins acting as an enzyme and an effector was previously proposed for a couple of PDEs, YciR (also called PdeR) (33) and PdeL (60). Such bifunctionality of a DGC/PDE, now seen with YfiN, would achieve local specificity of c-di-GMP signaling, since the second function is restricted to distinct downstream targets by their specific spatial organization. All of these examples share a requirement for enzymatic activity for their dual action, but YfiN requires an additional input signal, envelope stress, to serve its second function of cell division control. While we accidentally unearthed the distinct responses of YfiN to different stimuli, such multitasking may be common in c-di-GMP signaling, enabling bacteria to respond differentially to multiple environmental challenges.

### YfiN as a cell division checkpoint for adaptation to envelope stress.

From bacteria to eukaryotes, cell division regulation is used as a checkpoint to ensure survival upon exposure to stress. In bacteria, DNA damage (28) or envelope stress caused by inactivation of the peptidoglycan synthase PBP 3 (29) triggers the SOS response, in which the expression of the cell division inhibitor SulA is activated. While SulA directly inhibits FtsZ polymerization and leads to disassembly of the Z ring (39), YfiN retains the Z ring at the midcell (Fig. 2E and 3A). This feature of YfiN as a cell division inhibitor can also be observed with two DNA damage-induced proteins, SidA and DidA, in *C. crescentus*, a bacterium that does not have a SulA homolog. SidA and DidA arrest cell division by inhibiting late cell division events while retaining the Z ring (61, 62). Unlike SidA and DidA, however, cells with YfiN at the midcell have no visible constriction site (Fig. 2A), suggesting that YfiN inhibits the initiation of constriction. Although precisely which step in cell division is blocked by YfiN is not yet clear, exclusion of the late division protein FtsN and stalled septal PG synthesis at the future division sites occupied by YfiN raise the possibility that YfiN inhibits the initiation of constriction by using early division proteins as docking sites and preventing the recruitment of late division proteins (Fig. 2F and G). It has been reported that the interplay between FtsN and two early division proteins, FtsA and ZipA, is essential for the activation of constriction (41, 42), which might be affected by YfiN. We also note that some cells expressing YfiN_GFP_ show wide rings at the midcell, which are often slanted or off center (Fig. 4 to 6; see also Fig. S5 to S6 in the supplemental material), reminiscent of FtsZ structures seen under several conditions, including mutations in FtsZ (63, 64), overexpression of FtsZ (65, 66), overexpression of the FtsZ polymerization regulator ZapA (67), and the absence of low-molecular-weight penicillin-binding proteins (LMW PBPs) (66). It is possible that the observed aberrant localization of YfiN_GFP_ is due to altered polymerization of FtsZ.

Another unusual (and thus-far unique) feature of YfiN as a cell division inhibitor is that, unlike the known division inhibitors SulA (68), SidA (61), and DidA (62), cell division arrest by YfiN only leads to a modest level of cell lengthening and does not lead to filamentation (see Fig. S8 in the supplemental material). This suggests that YfiN may inhibit the synthesis of nascent PG not only at the division site but also along the lateral wall. Rod-shaped bacteria are thought to have two modes of cell wall synthesis catalyzed by different PG synthases: one responsible for cell elongation along the lateral wall and the other for the formation of the division septum (69). Our results suggest that YfiN might target a common step that these two modes share. Unlike the general inhibition of PG synthesis by β-lactam antibiotics (70, 71), the action of YfiN does not trigger cell lysis, as judged by the observation that cell density did not decrease when cell division was arrested by YfiN (Fig. 2E and 3A).

Given the positive effect of YfiN on survival under lethal PMB exposure (Fig. 7), it is tempting to speculate that the effector function of YfiN reported in this study is an adaptation mechanism that delays cell division and new peptidoglycan synthesis when *E. coli* cells experience envelope-disrupting environments, ensuring that the cell wall is not disarranged while the cell is recovering from the stress. Further investigation of YfiN will provide new insights into the mechanism by which bacterial cell division and cell wall synthesis are coordinated in response to environmental stress.

## MATERIALS AND METHODS

### **Strains, growth conditions, mutagenesis**, **and plasmid constructions.**

The strains and plasmids used in this study are listed in Table S1 in the supplemental material. The wild-type parent strains for *S. enterica*, *E. coli*, and *P. aeruginosa* were strains 14028, MG1655, and PAO1, respectively. All strains were grown in LB broth (10 g/liter tryptone, 5 g/liter yeast extract, 5 g/liter NaCl). When appropriate, the following antibiotics were used: ampicillin (100 µg/ml), chloramphenicol (20 µg/ml), kanamycin (50 µg/ml), and gentamicin (30 µg/ml). For inducible plasmids, isopropyl-β-d-thiogalactopyranoside (IPTG) and l-arabinose were added as indicated in the figures or figure legends.

Mutants of *Salmonella* and *E. coli* were constructed by inserting a kanamycin resistance cassette into the designated gene as previously described (72). Excision of the inserted cassettes was achieved by expression of the FLP recombinase encoded on pCP20 (72). The resulting strains were confirmed by DNA sequencing. Mutant combinations were prepared by P22 transduction.

For cloning of expression plasmids, gene sequences were amplified from the genomic DNA of wild-type strains by using PCR and introduced into pBAD30, pBAD33, pTrc99A, and pJN105. For DgcA expression vectors, pAB551, a gift from U. Jenal (40), was used as a template. Fusion proteins and an active-site mutant of YfiN were constructed using overlap extension PCR. All the resulting constructs were confirmed by DNA sequencing.

### Swimming motility assay.

LB swim plates were made using 0.3% Bacto agar. Plates were inoculated with 5 µl of an overnight culture in the center and incubated at 37°C for 8 h.

### Fluorescence microscopy.

Overnight cultures of cells with plasmids encoding fluorescent fusion proteins were diluted 1:100 in fresh LB medium with antibiotics and grown at 30°C with 0.005% arabinose for 4 h (unless otherwise stated). For imaging cells under no stress, a cell suspension (80 µl) was applied to a polylysine-coated slide, incubated for 15 min, and washed with LB medium (80 µl) before imaging. For imaging cells under stress (mostly *E. coli*), after 15 min of incubation, cells were washed, treated with LB containing the indicated stress-causing agent (80 µl), and incubated for 30 min before imaging. All slides for microscopy were prepared at room temperature. Stress-induced relocation was not dependent on immobilization by polylysine since it was also observed when stressors were directly added to broth cultures. For the *E. coli* temperature-sensitive mutants, after 30 min of stress exposure at room temperature, cells were incubated for another 30 min at the indicated temperature (30°C or 42°C) before microscopy. Images were acquired using an Olympus BX53 microscope, appropriate filters, and cellSens standard software (version 1.6) from Olympus and minimally processed using Adobe Photoshop 11.0.

### HADA labeling.

Nascent peptidoglycan synthesis was probed by the fluorescent d-amino acid HADA (purchased from *M. van* Nieuwenhze at Indiana University) as described previously (43). *Salmonella enterica* strain 14028 Δ*yfiN* bearing pBAD33 carrying ^S^*yfiN-yfp* was grown at 30°C for 4 h with 0.2% glucose or 0.005% arabinose, and HADA was added to a final concentration of 500 µM. After 1 min of incubation at 30°C, cells were fixed in ice-cold 70% ethanol and incubated on ice for 15 min. The fixed cells were washed and resuspended in phosphate-buffered saline and then imaged on 1% agarose pads (43).

### Bacterial two-hybrid assay.

To construct plasmids used for BACTH analysis (73), gene sequences (*yfiN*, *ftsZ*, *ftsA*, *zipA*, and *mreB*) were amplified from the genomic DNA of wild-type *E. coli* MG1655 by using PCR. The amplified DNA fragments were introduced between the HindIII and XbaI sites (*yfiN*, *ftsZ*, and *zipA*) or the XbaI and SacI sites (*ftsA*) of pUT18 and pKNT25 vectors or between the XbaI and BamHI sites (*mreB*) of pUT18C. The *E. coli* K-12 strain XL1-Blue (Stratagene) was used in all of the cloning steps, and the DNA sequences of the constructs were verified by sequencing.

For interaction analysis, plasmid combinations of pUT18(C) and pKNT25 were cotransformed into the *E. coli* strain BTH101. Five-microliter amounts of overnight cultures of transformants were spotted onto LB agar plates supplemented with ampicillin, kanamycin, IPTG (0.5 mM), and 40 µg/ml 5-bromo-4-chloro-3-indolyl-β-d-galactopyranoside (X-Gal). For analysis of c-di-GMP-stimulated interactions, chloramphenicol and arabinose (0.2%) were additionally added to the LB agar plates. Images of the plates were taken after 36 h of incubation at 30°C.

### Quantification of c-di-GMP.

The c-di-GMP concentrations were measured following a previously reported method (74). *E. coli* MG1655 wild type and MG1655 carrying pBAD30-^E^YfiN_GFP_ were grown at 30°C for 4 h in 5 ml LB supplemented with 0.005% arabinose (the optical density at 600 nm reached around 1.2). Intracellular nucleotides were extracted with a mixture of acetonitrile-methanol-water (40:40:20, vol/vol/vol) as described previously (74). Samples were analyzed by high-performance liquid chromatography–tandem mass spectrometry (HPLC-MS/MS) at the Metabolomics Core Facility at the University of Texas Health Science Center at San Antonio, using a Thermo Fisher Q Exactive mass spectrometer with online separation by a Thermo Fisher Dionex UltiMate 3000 HPLC instrument. As described previously (74), 0.1% (vol/vol) acetic acid in water with 10 mM ammonium acetate was used as LC solvent A, and solvent B was methanol. For a standard curve, c-di-GMP and xanthosine 3′,5′-cyclic monophosphate (cXMP) purchased from Axxora LLC (San Diego, CA) were used. As an internal standard, 1 µM cXMP was added to the extract. The result showed that the intracellular c-di-GMP concentration of cells expressing ^E^YfiN_GFP_ was 14.46 µM, while that of wild-type cells was 0.23 µM, confirming the c-di-GMP-synthetic activity of ^E^YfiN_GFP_.

## SUPPLEMENTAL MATERIAL

Table S1  Strains, plasmids, and phage. Table S1, PDF file, 0.2 MB

Figure S1  Identification of diguanylate cyclases (DGCs) and phosphodiesterases (PDEs) that contribute to motility. Swimming motility of *Salmonella enterica* wild type (strain 14028) and of mutants with single-gene knockouts of GGDEF/EAL domain proteins in the wild type (A) and in the Δ*yhjH* strain (B). Overnight cultures of each strain were inoculated at the center of 0.3% agar swim plates and incubated at 37°C for 8 h. Error bars indicate standard deviations of the results from four experimental repeats. In the Δ*yhjH* background (grey bars), mutations in two GGDEF domain proteins, YfiN and STM4551, and two EAL domain proteins, YjcC and YfeA, resulted in enhanced or impaired motility, respectively, suggesting that the activity of these four proteins contributes to the regulation of motility in *Salmonella*. Fluorescent-protein fusions to all five proteins that affected motility, YhjH, YfiN, STM4551, YjcC, and YfeA, were constructed. Of these, only YfiN_GFP_ showed a distinct localization (Fig. 2A). Download Figure S1, PDF file, 0.2 MB

Figure S2  YfiN-GFP fusions are functional. Swimming motility of strains expressing YfiN variants or DgcA from plasmids. The motility assay reports on the functionality of a DGC because c-di-GMP inhibits motility in both *Salmonella* and *E. coli*. (A) *Salmonella* Δ*yfiN* transformed with a plasmid encoding ^S^YfiN, ^S^YfiN_GFP_, or ^S^YfiN(GGAAF)_GFP_. The data show that the GFP fusion protein is just as active as native YfiN and that the GGDEF active-site motif is required for inhibition of motility. (B) *E. coli* Δ*yfiN* transformed with a plasmid encoding ^E^YfiN_GFP_, ^P^YfiN_GFP_, or DgcA from *Caulobacter crescentus*. All three DGCs are functional. Growth conditions were as described in the legend to Fig. S1. A concentration of 0.2% arabinose was used for expression from the P_BAD_ promoter and 0.1 mM IPTG for expression from the P_trc_ promoter. Download Figure S2, PDF file, 0.2 MB

Figure S3  YfiN midcell localization is independent of MreB. (A) ^S^YfiN_GFP_ localization in the absence and presence of A22, an inhibitor of MreB. *Salmonella* Δ*yfiN* cells expressing ^S^YfiN_GFP_ were treated with 5 µg/ml A22 for the indicated times. While cell morphology was affected by A22, this inhibitor did not alter the midcell localization of YfiN. Scale bar, 3 µm. (B) BACTH analysis with ^E^MreB and ^E^YfiN. ^E^YfiN did not show interaction with ^E^MreB, while direct interaction between ^E^MreB and ^E^FtsZ was observed, as previously reported (52). PC, positive control (T18-leucine zipper/T25-leucine zipper); NC, negative control (T18/T25 empty vectors). Download Figure S3, PDF file, 0.1 MB

Figure S4  Effects of various envelope-targeting stress conditions on ^E^YfiN localization. *E. coli* Δ*yfiN* cells expressing ^E^YfiN_GFP_ were grown in LB with inducer at 30°C for 4 h and then exposed to the indicated stress condition in LB for 30 min. The numbers at the bottom indicate the percentages of cells showing ^E^YfiN_GFP_ at the midcell. (A) Osmolality stress. Cells were exposed to NaCl stress, down- or upshifted as indicated by the arrows. (B) OM permeabilization stress. Cells were exposed to the indicated concentrations of PMB in LB. (C) Results obtained using other cell envelope stressors, including ampicillin, sodium dodecyl sulfate (SDS), and lysozyme. None of these envelope stressors induced ^E^YfiN relocation. Since pBAD30 carries an ampicillin-resistant gene, pBAD33 was used for expression in the experiment whose results are shown in this panel. Scale bar, 3 µm. Download Figure S4, PDF file, 0.4 MB

Figure S5  ^E^YfiN relocation to the midcell is not affected by the presence of the protein synthesis inhibitor chloramphenicol (CM). *E. coli* Δ*yfiN* cells expressing ^E^YfiN_GFP_ were exposed to no stress, 250 mM NaCl, and 250 mM NaCl in the presence of CM (300 µg/ml). Scale bar, 3 µm. Download Figure S5, PDF file, 0.1 MB

Figure S6  Localization of ^E^YfiN in the wild type and temperature-sensitive mutants with mutations of *E. coli* cell division proteins. For each strain, cells producing ^E^YfiN_GFP_ were exposed to 10 mM EDTA for 30 min and incubated for another 30 min at two different temperatures, 30°C and 42°C, before imaging. ^E^YfiN lost its midcell localization in the absence of ^E^FtsZ or ^E^ZipA. Scale bar, 3 µm. Download Figure S6, PDF file, 0.1 MB

Figure S7  ^P^YfiN does not localize to the midcell in *P. aeruginosa*, *Salmonella*, or *E. coli*. For each strain, after 4 h of growth, cells expressing ^P^YfiN_GFP_ were photographed before and 30 min after osmotic upshift (250 mM NaCl). Exposure to the stress is indicated by a thunderbolt. ^P^YfiN failed to localize to the midcell regardless of the host strain. Scale bar, 3 µm. Download Figure S7, PDF file, 0.1 MB

Figure S8  Cell division arrest by YfiN does not lead to cell filamentation. Cell morphology of *Salmonella* 14028 wild type expressing ^S^SulA and ^S^YfiN_GFP_. Cells were grown with 0.2% glucose at 30°C for 4 h and then washed and resuspended in LB with 0.005% arabinose for 2 h of induction. Unlike the results seen with SulA, cells did not filament with YfiN. Images were taken before and 2 h after adding arabinose. Scale bar, 3 µm. Download Figure S8, PDF file, 0.1 MB
