# Supplementary material for: A Diguanylate Cyclase Acts as a Cell Division Inhibitor in a Two-Step Response to Reductive and Envelope Stresses
Source: mBio. 2016 Aug 9;7(4):e00822-16. doi: 10.1128/mBio.00822-16 (PMC4992967; doi:10.1128/mBio.00822-16)
Supplement: Table S1 — Strains, plasmids, and phage. [file mbo004162924st1.pdf]

Table S1. Strains, Plasmids and Phage

Strain, Plasmid, Phage	Genotype or Description	Reference
S. enterica		
14028	wild type ATCC strain	(1)
HK132	Δ*yfiN*; 14028 with deletion of *yfiN*	This study
QW262	Δ*yhjH*	(2)
HK133	Δ*STM1987*	This study
HK134	Δ*yeaJ*	This study
HK135	Δ*STM4551*	This study
HK136	Δ*adrA*	This study
HK137	Δ*yjcC*	This study
HK138	Δ*ylaB*	This study
HK139	Δ*STM1827*	This study
HK140	Δ*STM0343*	This study
HK141	Δ*STM2215*	This study
HK142	Δ*STM3388*	This study
HK143	Δ*yciR*	This study
HK144	Δ*yegE*	This study
HK145	Δ*yhdA*	This study
HK147	Δ*ydiV*	This study
HK148	Δ*STM2503*	This study
HK149	Δ*yhjK*	This study
HK150	Δ*yfeA*	This study
HK164	Δ*yhjH* Δ*yfiN*; 14028 with double deletions of *yhjH* and *yfiN*	This study
HK165	Δ*yhjH* Δ*STM1987*	This study
HK166	Δ*yhjH* Δ*yeaJ*	This study
HK167	Δ*yhjH* Δ*STM4551*	This study
HK168	Δ*yhjH* Δ*adrA*	This study
HK169	Δ*yhjH* Δ*yjcC*	This study
HK170	Δ*yhjH* Δ*ylaB*	This study
HK171	Δ*yhjH* Δ*STM1827*	This study
HK172	Δ*yhjH* Δ*STM0343*	This study
HK173	Δ*yhjH* Δ*STM2215*	This study
HK174	Δ*yhjH* Δ*STM3388*	This study
HK175	Δ*yhjH* Δ*yciR*	This study
HK176	Δ*yhjH* Δ*yegE*	This study
HK177	Δ*yhjH* Δ*yhdA*	This study
HK178	Δ*yhjH* Δ*ydiV*	This study
HK179	Δ*yhjH* Δ*STM2503*	This study
HK180	Δ*yhjH* Δ*yhjK*	This study
HK181	Δ*yhjH* Δ*yfeA*	This study
HK185	HK164 + pBAD30	This study
HK186	HK164 + pBAD30-^{S}YfiN	This study
HK187	HK164 + pBAD30-^{S}YfiN(GGAAF; active site mutant)	This study
HK261	HK132 + pBAD30-^{S}YfiN$_{GFP}$	This study
HK620	HK132 + pBAD33-^{S}YfiN(GGAAF)$_{GFP}$	This study

HK435	HK132 + pBAD30-^{S}YfiN	This study
HK269	HK132 + pBAD30-DgcA	This study
HK375	HK132 + pBAD33-^{S}YfiN$_{YFP}$ + pBAD30-^{S}FtsA$_{CFP}$	This study
HK373	HK261+ pBAD33	This study
HK372	HK261 + pBAD33-^{S}SulA	This study
HK683	HK132 + pBAD33-^{S}YfiN$_{YFP}$ + pBAD30-$_{CFP}$^{S}FtsN	This study
HK369	HK132 + pBAD33-^{S}YfiN$_{YFP}$	This study
HK377	HK132 + pBAD30-^{P}YfiN$_{GFP}$	This study
HK698	14028 + pBAD33-^{S}SulA	This study
HK631	14028 + pBAD30- ^{S}YfiN$_{GFP}$	This study
E. coli		
MG1655	K12 wild type strain: F^-, λ^-, *rph*-1	Laboratory Collection
HK359	Δ*yfiN*; MG1655 with deletion of *yfiN*	This study
HK360	Δ*yfiB*; MG1655 with deletion of *yfiB*	This study
HK361	Δ*yfiR*; MG1655 with deletion of *yfiR*	This study
WM1125	MG1655 *lacU169 ftsZ84*	(3)
WM1115	MG1655 *lacU169 ftsA12*	(4)
PS223	W3110 *zipA1*	(5)
JW3832	BW25113 *dsbA::kan*	(6)
BTH101	F^-,*cya*-99, *ara*D139, *gal*E15, *gal*K16, *rps*L1, *hsd*R2, *mcr*A1, *mcr*B1	(7)
XL1-blue	*RecA1, endA1, gyrA96, thi-1, hsdR17, supE44, relA1, lac,* [F′, *proAB, lac1qZΔM15, Tn10 (tet′)*]	Stratagene
HK365	HK359 + pBAD30-^{E}YfiN$_{GFP}$	This study
HK366	HK360 + pBAD30-^{E}YfiN$_{GFP}$	This study
HK549	HK359 + pBAD33-^{E}YfiN$_{GFP}$	This study
HK489	MG1655 *yfiN::* ^{E}*yfiN-gfp*	This study
HK531	MG1655 *yfiR::kan*(←)	This study
HK532	MG1655 *yfiR::kan*(←) *yfiN*::P$_{BAD}$-^{E}*yfiN-gfp*	This study
HK604	MG1655 + pBAD30-DgcA	This study
HK635	MG1655 + pBAD30-^{E}YfiN$_{GFP}$	This study
HK381	WM1125 + pBAD30-^{E}YfiN$_{GFP}$	This study
HK380	WM1115 + pBAD30-^{E}YfiN$_{GFP}$	This study
HK590	PS223 + pBAD30-^{E}YfiN$_{GFP}$	This study
HK470	BTH101 + pUT18 + pKNT25	This study
HK469	BTH101 + pUT18-zip + pKNT25-zip	This study
HK597	BTH101 + pUT18-^{E}YfiN + pKNT25-^{E}YfiN	This study
HK598	BTH101 + pUT18-^{E}YfiN + pKNT25-^{E}FtsZ	This study
HK599	BTH101 + pUT18-^{E}YfiN + pKNT25-^{E}FtsA	This study
HK600	BTH101 + pUT18-^{E}YfiN + pKNT25-^{E}ZipA	This study
HK725	BTH101 + pUT18C-^{E}MreB + pKNT25- ^{E}FtsZ	This study
HK726	BTH101 + pUT18C-^{E}MreB + pKNT25- ^{E}YfiN	This study
HK636	HK359 + pBAD30-^{P}YfiN$_{GFP}$	This study
HK571	HK359 + pBAD33-^{E}YfiN(GGAAF)$_{GFP}$ + pTrc99A	This study
HK572	HK359 + pBAD33-^{E}YfiN(GGAAF)$_{GFP}$ + pTrc99A-DgcA	This study

HK580	BTH101 + pBAD33 + pUT18-^{E}YfiN(GGAAF) + pKNT25-^{E}FtsZ	This study
HK582	BTH101 + pBAD33 + pUT18-^{E}YfiN(GGAAF) + pKNT25	This study
HK576	BTH101 + pBAD33-DgcA + pUT18-^{E}YfiN(GGAAF) + pKNT25-^{E}FtsZ	This study
HK578	BTH101 + pBAD33-DgcA + pUT18-^{E}YfiN(GGAAF) + pKNT25	This study
HK608	BTH101 + pBAD33 + pUT18-^{E}YfiN(GGAAF) + pKNT25-^{E}ZipA	This study
HK609	BTH101 + pBAD33 + pUT18-^{E}YfiN(GGAAF) + pKNT25	This study
HK606	BTH101 + pBAD33-DgcA + pUT18-^{E}YfiN(GGAAF) + pKNT25-^{E}ZipA	This study
HK607	BTH101 + pBAD33-DgcA + pUT18-^{E}YfiN(GGAAF) + pKNT25	This study
HK551	HK359 + pBAD33-^{E}YfiN$_{GFP}$ + pTrc99A	This study
HK552	HK359 + pBAD33-^{E}YfiN$_{GFP}$ + pTrc99A-^{E}YfiR	This study
HK367	HK361 + pBAD30-^{E}YfiN$_{GFP}$	This study
HK626	JW3832 + pBAD33-^{E}YfiN$_{GFP}$ + pTrc99A-^{E}YfiR	This study
P. aeruginosa		
PAO1	PAO1 wild type	gift from M. Whiteley
HK376	PAO1 + pJN105-^{P}YfiN$_{GFP}$	This study
**Plasmid**		
pKD4	Kanamycin resistance gene template	(8)
pKD46	λ Red Recombinase	(8)
pCP20	FLP recombinase	(8)
pBAD30	Cloning vector; P$_{BAD}$ and Amp^{R}	(9)
pBAD33	Cloning vector; P$_{BAD}$ and Cm^{R}	(9)
pTrc99A	Cloning vector; P$_{Trc}$ and Amp^{R}	(10)
pJN105	Cloning vector; P$_{BAD}$ and Gm^{R}	(11)
pAB551	P$_{BAD}$::*dgcA* from *C. crescentus*	(12)
pBAD30-^{S}YfiN	P$_{BAD}$:: ^{S}*yfiN*	This study
pBAD30-^{S}YfiN(GGAAF)	P$_{BAD}$::^{S}*yfiN*(D327A E328A)	This study
pBAD30-^{S}YfiN$_{GFP}$	P$_{BAD}$:: ^{S}*yfiN-gfp*	This study
pBAD33-^{S}YfiN(GGAAF)$_{GFP}$	P$_{BAD}$:: ^{S}*yfiN*(D327A E328A)-*gfp*	This study
pBAD33-^{S}YfiN$_{YFP}$	P$_{BAD}$:: ^{S}*yfiN-yfp*	This study
pBAD30-^{S}FtsA$_{CFP}$	P$_{BAD}$:: ^{S}*ftsA-cfp*	This study
pBAD33-^{S}SulA	P$_{BAD}$:: ^{S}*sulA*	This study
pBAD30-^{S}$_{CFP}$FtsN	P$_{BAD}$::*cfp*-^{S}*ftsN*	This study
pBAD30-^{E}YfiN$_{GFP}$	P$_{BAD}$:: ^{E}*yfiN-gfp*	This study
pUT18	P$_{lac}$::-*T18*; Amp^{R}	(7)
pUT18C	P$_{lac}$::*T18*-; Amp^{R}	(7)
pKNT25	P$_{lac}$::-*T25*; Kan^{R}	(7)
pUT18-zip	P$_{lac}$:: leucine zipper region from yeast GCN4-*T18*	(7)
pKNT25-zip	P$_{lac}$:: leucine zipper region from yeast GCN4-*T25*	(7)
pUT18-^{E}YfiN	P$_{lac}$:: ^{E}*yfiN-T18*	This study

pUT18-^{E}FtsZ	P$_{lac}$:: ^{E}*ftsZ-T18*	This study
pUT18-^{E}FtsA	P$_{lac}$:: ^{E}*ftsA-T18*	This study
pUT18-^{E}ZipA	P$_{lac}$:: ^{E}*zipA-T18*	This study
pKNT25-^{E}YfiN	P$_{lac}$:: ^{E}*yfiN-T25*	This study
pUT18C-^{E}MreB	P$_{lac}$::*T18-*^{E}*mreB*	This study
pBAD33-^{E}YfiN(GGAAF)$_{GFP}$	P$_{BAD}$::^{E}*yfiN* (D329A E330A)-*gfp*	This study
pTrc99A-DgcA	P$_{trc}$::*dgcA* (amplified from pAB551)	This study
pUT18-^{E}YfiN(GGAAF)	P$_{lac}$:: ^{E}*yfiN* (D329A E330A)-*T18*	This study
pBAD30-DgcA	P$_{BAD}$::*dgcA*	This study
pBAD33-DgcA	P$_{BAD}$::*dgcA*	This study
pBAD33-^{E}YfiN$_{GFP}$	P$_{BAD}$:: ^{E}*yfiN-gfp*	This study
pTrc99A-^{E}YfiR	P$_{trc}$:: ^{E}*yfiR*	This study
pJN105-^{P}YfiN$_{GFP}$	P$_{BAD}$:: ^{P}*yfiN-gfp*	This study
pBAD30-^{P}YfiN$_{GFP}$	P$_{BAD}$:: ^{P}*yfiN-gfp*	This study
**Phage**		
P22	HT12/4int103	[1]

References

1. **Mariconda S, Wang Q, Harshey RM.** 2006. A mechanical role for the chemotaxis system in swarming motility. Mol Microbiol **60:**1590-1602.
2. **Wang Q, Mariconda S, Suzuki A, McClelland M, Harshey RM.** 2006. Uncovering a large set of genes that affect surface motility in Salmonella enterica serovar Typhimurium. J Bacteriol **188:**7981-7984.
3. **Yu XC, Margolin W.** 2000. Deletion of the min operon results in increased thermosensitivity of an ftsZ84 mutant and abnormal FtsZ ring assembly, placement, and disassembly. J Bacteriol **182:**6203-6213.
4. **Geissler B, Elraheb D, Margolin W.** 2003. A gain-of-function mutation in ftsA bypasses the requirement for the essential cell division gene zipA in Escherichia coli. Proc Natl Acad Sci U S A **100:**4197-4202.
5. **Pichoff S, Lutkenhaus J.** 2002. Unique and overlapping roles for ZipA and FtsA in septal ring assembly in Escherichia coli. EMBO J **21:**685-693.
6. **Baba T, Ara T, Hasegawa M, Takai Y, Okumura Y, Baba M, Datsenko KA, Tomita M, Wanner BL, Mori H.** 2006. Construction of Escherichia coli K-12 in-frame, single-gene knockout mutants: the Keio collection. Mol Syst Biol **2:**2006 0008.
7. **Karimova G, Pidoux J, Ullmann A, Ladant D.** 1998. A bacterial two-hybrid system based on a reconstituted signal transduction pathway. Proc Natl Acad Sci U S A **95:**5752-5756.
8. **Datsenko KA, Wanner BL.** 2000. One-step inactivation of chromosomal genes in Escherichia coli K-12 using PCR products. Proc Natl Acad Sci U S A **97:**6640-6645.
9. **Guzman LM, Belin D, Carson MJ, Beckwith J.** 1995. Tight regulation, modulation, and high-level expression by vectors containing the arabinose PBAD promoter. J Bacteriol **177:**4121-4130.
10. **Amann E, Brosius J, Ptashne M.** 1983. Vectors bearing a hybrid trp-lac promoter useful for regulated expression of cloned genes in Escherichia coli. Gene **25:**167-178.
11. **Newman JR, Fuqua C.** 1999. Broad-host-range expression vectors that carry the L-arabinose-inducible Escherichia coli araBAD promoter and the araC regulator. Gene **227:**197-203.
12. **Steiner S, Lori C, Boehm A, Jenal U.** 2013. Allosteric activation of exopolysaccharide synthesis through cyclic di-GMP-stimulated protein-protein interaction. EMBO J **32:**354-368.
