# Supplementary figures and images for: A Diguanylate Cyclase Acts as a Cell Division Inhibitor in a Two-Step Response to Reductive and Envelope Stresses
Source: mBio. 2016 Aug 9;7(4):e00822-16. doi: 10.1128/mBio.00822-16 (PMC4992967; doi:10.1128/mBio.00822-16)

A

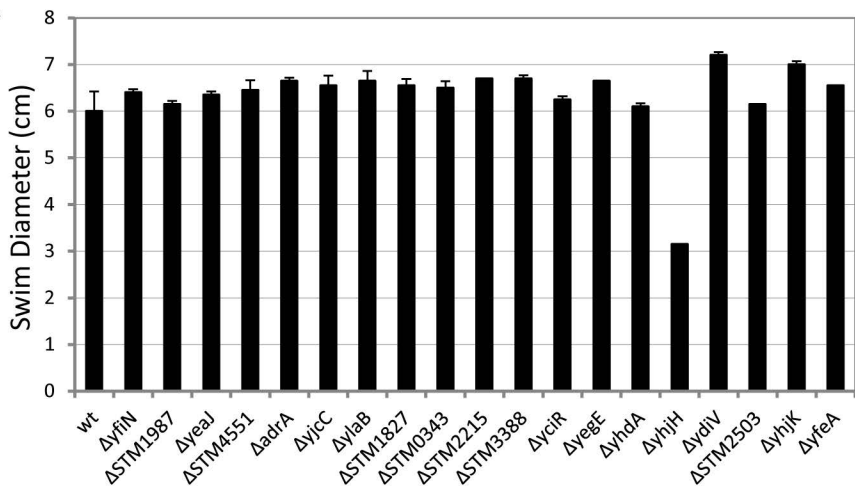

B

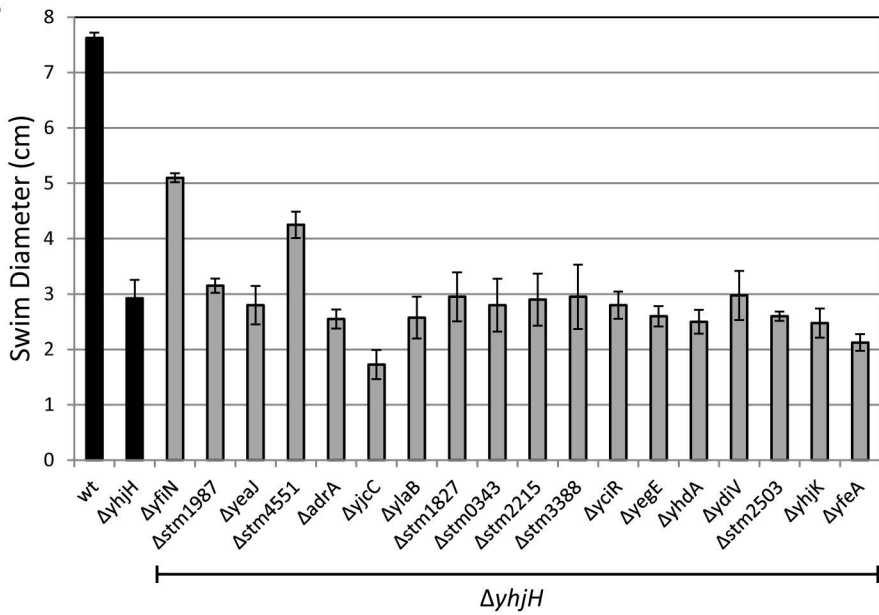

Supplement: Figure S1 — Identification of diguanylate cyclases (DGCs) and phosphodiesterases (PDEs) that contribute to motility. Swimming motility of Salmonella enterica wild type (strain 14028) and of mutants with single-gene knockouts of GGDEF/EAL domain proteins in the wild type (A) and in the ΔyhjH strain (B). Overnight cultures of each strain were inoculated at the center of 0.3% agar swim plates and incubated at 37°C for 8 h. Error bars indicate standard deviations of the results from four experimental repeats. In the ΔyhjH background (grey bars), mutations in two GGDEF domain proteins, YfiN and STM4551, and two EAL domain proteins, YjcC and YfeA, resulted in enhanced or impaired motility, respectively, suggesting that the activity of these four proteins contributes to the regulation of motility in Salmonella. Fluorescent-protein fusions to all five proteins that affected motility, YhjH, YfiN, STM4551, YjcC, and YfeA, were constructed. Of these, only YfiNGFP showed a distinct localization (Fig. 2A). Download [file mbo004162924sf1.pdf]

A

no A22

After 1 hr of A22 treatment

After 2 hrs of A22 treatment

Phase

 $^{\text{SYfiN}}_{\text{GFP}}$ 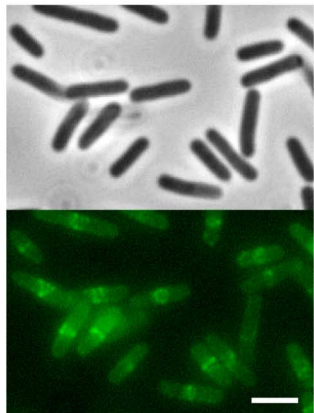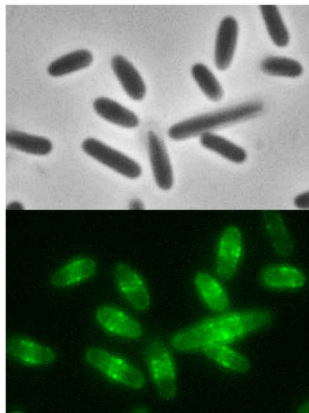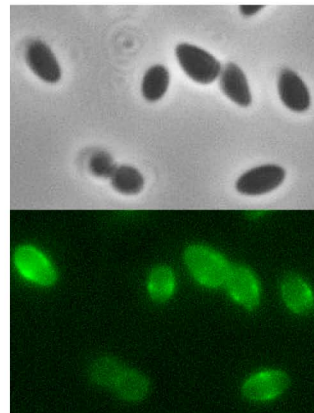

B

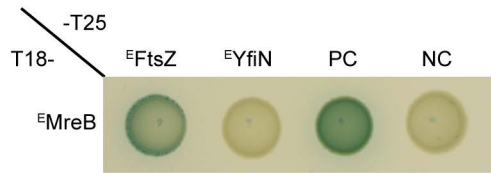

Supplement: Figure S3 — YfiN midcell localization is independent of MreB. (A) SYfiNGFP localization in the absence and presence of A22, an inhibitor of MreB. Salmonella ΔyfiN cells expressing SYfiNGFP were treated with 5 µg/ml A22 for the indicated times. While cell morphology was affected by A22, this inhibitor did not alter the midcell localization of YfiN. Scale bar, 3 µm. (B) BACTH analysis with EMreB and EYfiN. EYfiN did not show interaction with EMreB, while direct interaction between EMreB and EFtsZ was observed, as previously reported (52). PC, positive control (T18-leucine zipper/T25-leucine zipper); NC, negative control (T18/T25 empty vectors). Download [file mbo004162924sf3.pdf]

A

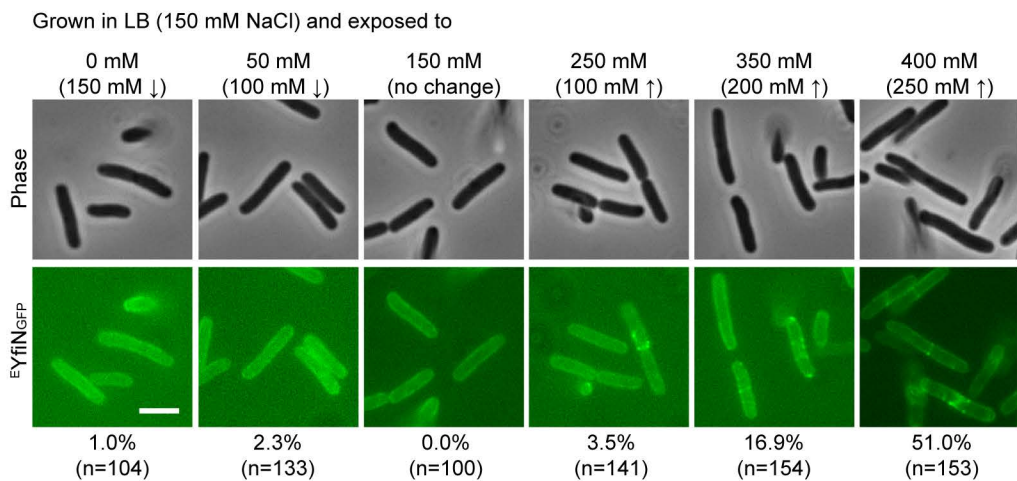

B

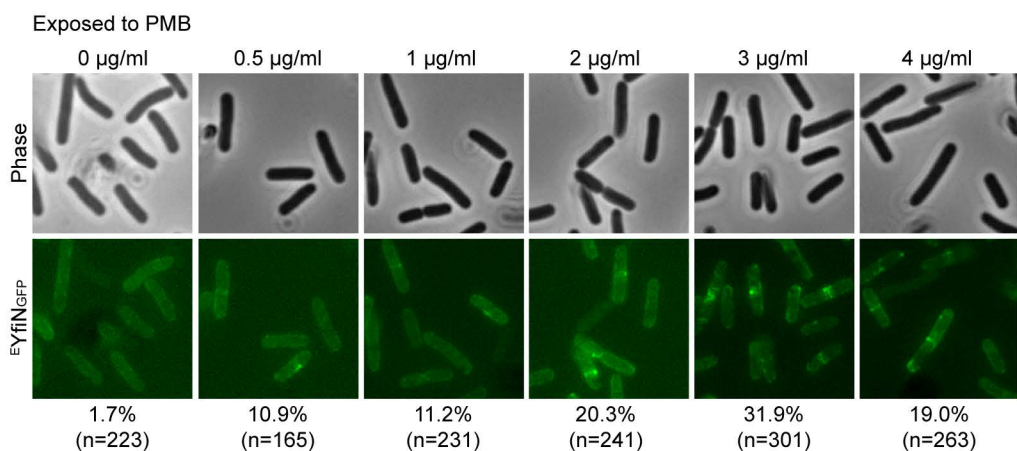

C

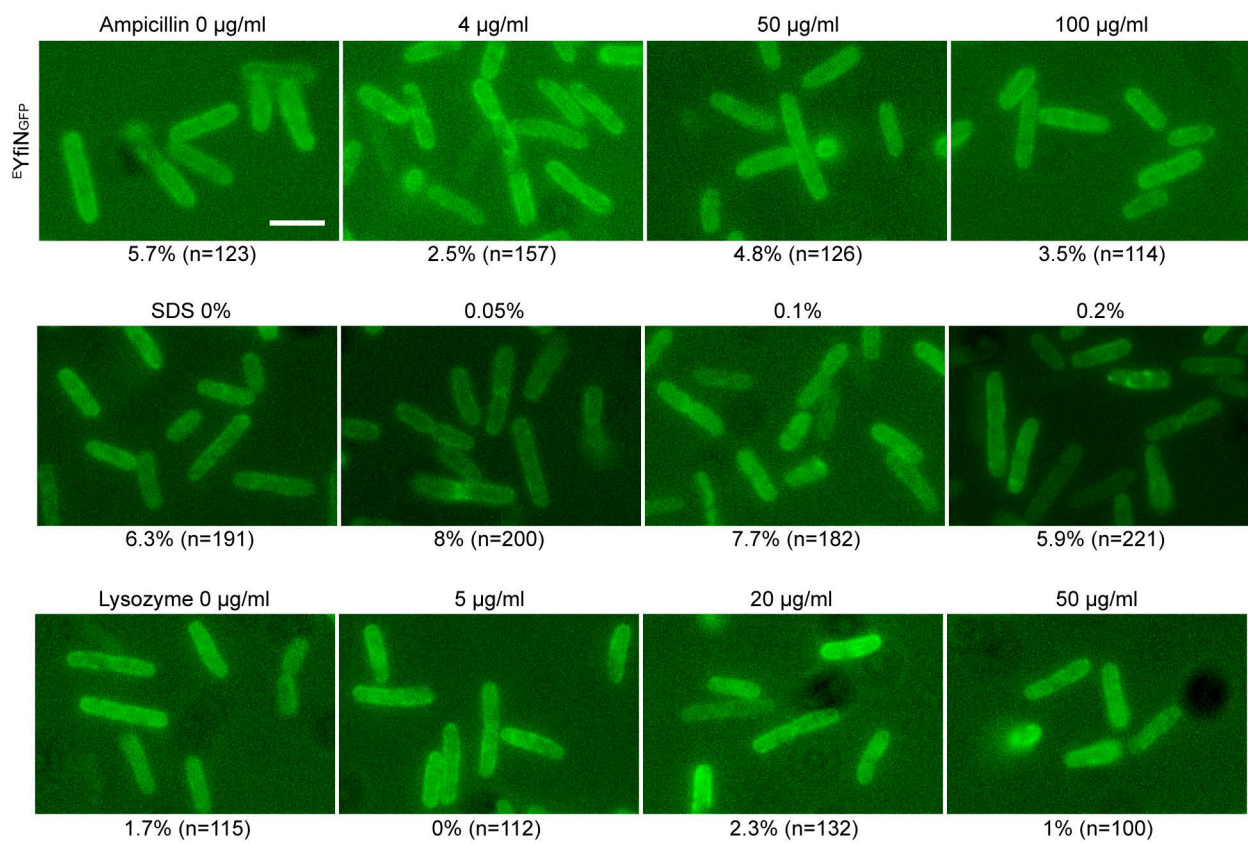

Supplement: Figure S4 — Effects of various envelope-targeting stress conditions on EYfiN localization. E. coli ΔyfiN cells expressing EYfiNGFP were grown in LB with inducer at 30°C for 4 h and then exposed to the indicated stress condition in LB for 30 min. The numbers at the bottom indicate the percentages of cells showing EYfiNGFP at the midcell. (A) Osmolality stress. Cells were exposed to NaCl stress, down- or upshifted as indicated by the arrows. (B) OM permeabilization stress. Cells were exposed to the indicated concentrations of PMB in LB. (C) Results obtained using other cell envelope stressors, including ampicillin, sodium dodecyl sulfate (SDS), and lysozyme. None of these envelope stressors induced EYfiN relocation. Since pBAD30 carries an ampicillin-resistant gene, pBAD33 was used for expression in the experiment whose results are shown in this panel. Scale bar, 3 µm. Download [file mbo004162924sf4.pdf]

No stress

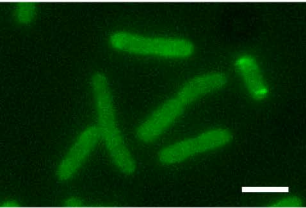

EYfiNGFP

NaCl stress

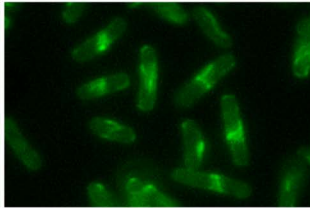

NaCl stress  
in the presence of CM

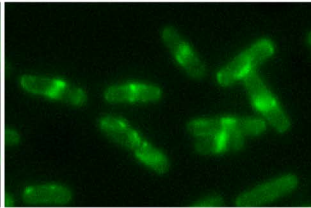

Supplement: Figure S5 — EYfiN relocation to the midcell is not affected by the presence of the protein synthesis inhibitor chloramphenicol (CM). E. coli ΔyfiN cells expressing EYfiNGFP were exposed to no stress, 250 mM NaCl, and 250 mM NaCl in the presence of CM (300 µg/ml). Scale bar, 3 µm. Download [file mbo004162924sf5.pdf]

wild type

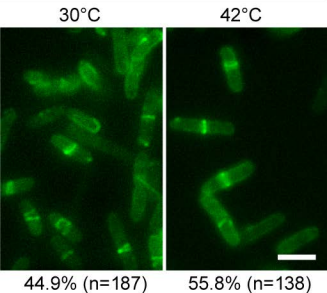

*ftsZts*

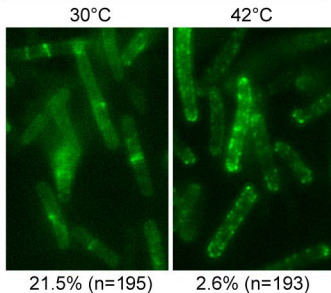

*ftsAts*

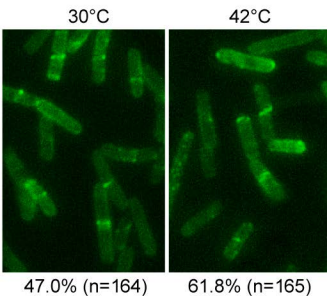

*zipAts*

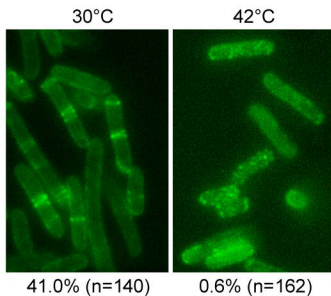

Supplement: Figure S6 — Localization of EYfiN in the wild type and temperature-sensitive mutants with mutations of E. coli cell division proteins. For each strain, cells producing EYfiNGFP were exposed to 10 mM EDTA for 30 min and incubated for another 30 min at two different temperatures, 30°C and 42°C, before imaging. EYfiN lost its midcell localization in the absence of EFtsZ or EZipA. Scale bar, 3 µm. Download [file mbo004162924sf6.pdf]

$P^{YfiN_{GFP}}$   
in *Salmonella*

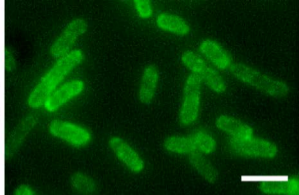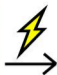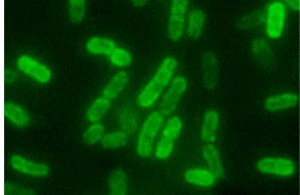

$P^{YfiN_{GFP}}$   
in *E. coli*

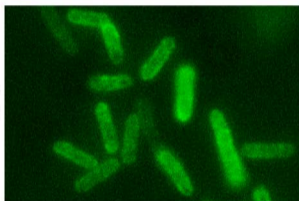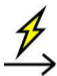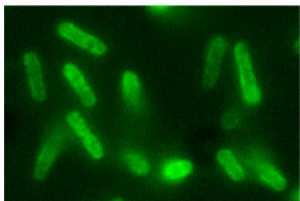

$P^{YfiN_{GFP}}$   
in *P. aeruginosa*

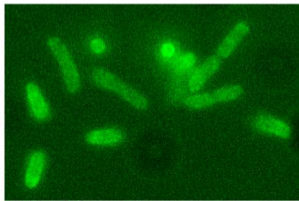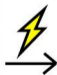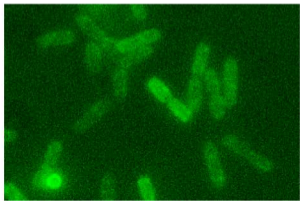

Supplement: Figure S7 — PYfiN does not localize to the midcell in P. aeruginosa, Salmonella, or E. coli. For each strain, after 4 h of growth, cells expressing PYfiNGFP were photographed before and 30 min after osmotic upshift (250 mM NaCl). Exposure to the stress is indicated by a thunderbolt. PYfiN failed to localize to the midcell regardless of the host strain. Scale bar, 3 µm. Download [file mbo004162924sf7.pdf]
