# Supplementary material for: A Diguanylate Cyclase Acts as a Cell Division Inhibitor in a Two-Step Response to Reductive and Envelope Stresses
Source: mBio. 2016 Aug 9;7(4):e00822-16. doi: 10.1128/mBio.00822-16 (PMC4992967; doi:10.1128/mBio.00822-16)
Supplement: Figure S2 — YfiN-GFP fusions are functional. Swimming motility of strains expressing YfiN variants or DgcA from plasmids. The motility assay reports on the functionality of a DGC because c-di-GMP inhibits motility in both Salmonella and E. coli. (A) Salmonella ΔyfiN transformed with a plasmid encoding SYfiN, SYfiNGFP, or SYfiN(GGAAF)GFP. The data show that the GFP fusion protein is just as active as native YfiN and that the GGDEF active-site motif is required for inhibition of motility. (B) E. coli ΔyfiN transformed with a plasmid encoding EYfiNGFP, PYfiNGFP, or DgcA from Caulobacter crescentus. All three DGCs are functional. Growth conditions were as described in the legend to Fig. S1. A concentration of 0.2% arabinose was used for expression from the PBAD promoter and 0.1 mM IPTG for expression from the Ptrc promoter. Download [file mbo004162924sf2.pdf]

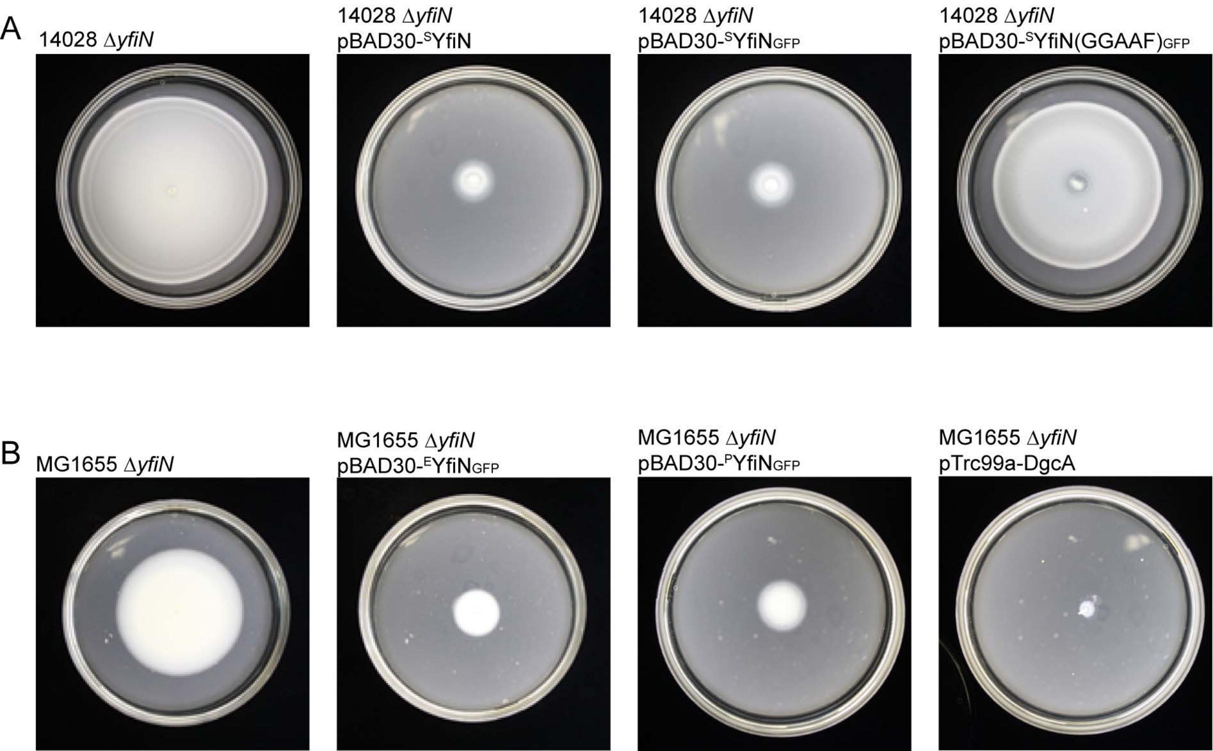
A
14028 ΔyfiN
14028 ΔyfiN
pBAD30-^{S}YfiN
14028 ΔyfiN
pBAD30-^{S}YfiN$_{GFP}$
14028 ΔyfiN
pBAD30-^{S}YfiN(GGAAF)$_{GFP}$
B
MG1655 ΔyfiN
MG1655 ΔyfiN
pBAD30-^{E}YfiN$_{GFP}$
MG1655 ΔyfiN
pBAD30-^{P}YfiN$_{GFP}$
MG1655 ΔyfiN
pTrc99a-DgcA
