# Supplementary material for: A Diguanylate Cyclase Acts as a Cell Division Inhibitor in a Two-Step Response to Reductive and Envelope Stresses
Source: mBio. 2016 Aug 9;7(4):e00822-16. doi: 10.1128/mBio.00822-16 (PMC4992967; doi:10.1128/mBio.00822-16)
Supplement: Figure S8 — Cell division arrest by YfiN does not lead to cell filamentation. Cell morphology of Salmonella 14028 wild type expressing SSulA and SYfiNGFP. Cells were grown with 0.2% glucose at 30°C for 4 h and then washed and resuspended in LB with 0.005% arabinose for 2 h of induction. Unlike the results seen with SulA, cells did not filament with YfiN. Images were taken before and 2 h after adding arabinose. Scale bar, 3 µm. Download [file mbo004162924sf8.pdf]

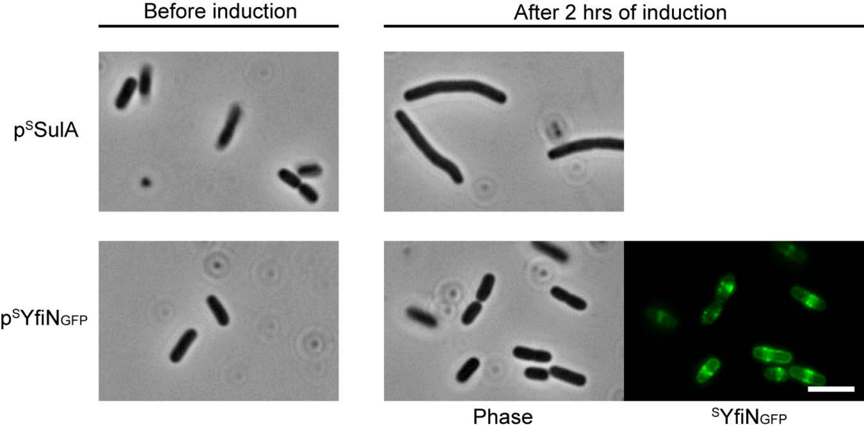

Before induction
After 2 hrs of induction
p^SSulA
p^SYfiN_GFP
Phase
^SYfiN_GFP
